# Examining the Effect of Virtual Reality–Based Fast-Food Marketing on Eating-Related Outcomes in Young Adults: Protocol for a Randomized Controlled Trial

**DOI:** 10.2196/69096

**Published:** 2025-09-22

**Authors:** Omni Cassidy, Emma Boyland, Susan Persky, Andrea B Troxel, Brian Elbel

**Affiliations:** 1 Division of Health and Behavior Department of Population Health NYU Grossman School of Medicine/Langone Health New York, NY United States; 2 Department of Psychology Institute of Population Health, University of Liverpool Liverpool United Kingdom; 3 Social and Behavioral Research Branch National Human Genome Research Institute National Institutes of Health Bethesda, MD United States; 4 Division of Biostatistics Department of Population Health NYU Grossman School of Medicine/Langone Health New York, NY United States; 5 Population Health and Health Policy NYU Wagner Graduate School of Public Service New York, NY United States

**Keywords:** virtual reality, digital food and beverage marketing, obesity, purchase intention, hunger

## Abstract

**Background:**

Black communities, compared to White communities, are disproportionately targeted with more unhealthy food advertisements on television and social media. Exposure to unhealthy food and beverage marketing is associated with appetitive sensations, purchase intention, and intake behaviors, which may contribute to poor overall diet quality and worsening nutritional disparities in Black communities. Despite the negative effects, food and beverage companies are expanding their reach and harnessing advanced technology to create immersive experiences using virtual reality (VR). Black young adults may be uniquely vulnerable.

**Objective:**

We aim to explore the effect of a VR-based fast-food marketing experience (compared with a VR-based nonfood control) on purchase intention, arousal, and hunger in a sample of Black and White young adults.

**Methods:**

We will recruit 200 Black and White young adults (aged 18-24 years) from the New York City metropolitan area for a 1-time, 2-hour laboratory-based study. After screening and obtaining informed consent, eligible participants will be randomized to 1 of 2 VR conditions: a VR-based fast-food marketing experience (Wendyverse; experimental) or a VR-based nonfood control (Nikeland). In the Wendyverse, users can order from the restaurant operated by Wendy’s, play games, meet others who may be visiting the Wendyverse, and access codes that can be used to obtain free food at physical restaurants. The control condition will be the Nikeland app, where participants can play sports, try on apparel, and engage with celebrity athletes. Study personnel will provide a 5-minute training session to participants before beginning the experiment to ensure that they feel comfortable in the VR environment. Participants will otherwise engage with the VR app independently. The primary outcomes will be fast-food purchase intention, assessed via a self-report questionnaire; arousal, assessed via electrodermal activity or skin conductance; and hunger, assessed via salivary reactivity. We will also conduct secondary analyses to examine interactions by race, ethnicity, and food or nutrition insecurity as a proxy for socioeconomic status. Analyses of covariance and multiple linear regressions will be conducted to examine the effects of VR-based fast-food marketing exposure on the relevant outcomes (compared to the control).

**Results:**

This study was funded by the National Institute on Minority Health and Health Disparities in September 2024. Recruitment is expected to begin in September 2025. We expect to complete data collection by October 2026 and begin data cleaning and analysis in November 2026.

**Conclusions:**

On the basis of previous research and data, we anticipate that young adults randomized to view VR-based food and beverage marketing will self-report higher purchase intention and demonstrate stronger arousal and hunger. The data will be used to support future research and improve the understanding of the effects of digital forms of unhealthy food and beverage marketing on young people.

**Trial Registration:**

ClinicalTrials.gov NCT06917391; https://clinicaltrials.gov/study/NCT06917391

**International Registered Report Identifier (IRRID):**

PRR1-10.2196/69096

## Introduction

### Background

Food and beverage companies disproportionately target Black consumers with unhealthy food and beverage marketing [1]. Exposure to unhealthy food and beverage marketing (hereafter referred to as “food marketing”) is powerful in shaping purchasing behaviors [2]. Exposure to food marketing contributes to stronger preferences for advertised foods [3-6], higher purchase intention [7], appetitive sensations [8], and increased reported [9-11] and observed intake of *both* advertised and nonadvertised foods [9,12-16]. Growing data also indicate that digital-based food and beverage marketing (eg, social media and gaming) is associated with stronger preferences for and increased intake of advertised foods [17-23].

Black young adults may be especially vulnerable to the effects of food marketing exposure [24,25]. Food marketing practices targeting Black communities, which are rooted in structural racism and discrimination [26,27], involve segmenting consumers based on geographic location, race, ethnicity, and income. Such marketing practices lead to 3 main issues. First, data on Black youth (aged 2-17 years) show that they have twice the exposure to unhealthy food advertisements compared to White youth [1]. Second, advertisements targeting Black consumers focus on less healthy products compared to advertisements targeting other audiences [1,28,29]. Third, advertisements targeting Black consumers are designed to be particularly powerful by (1) featuring racially and ethnically salient cues (eg, basketball references); (2) exploiting cost sensitivity due to income inequities present in some Black communities, particularly those who may be food or nutritionally insecure; and (3) capitalizing on Black consumers’ greater trust in and responsiveness to advertisements after a history of being excluded from the marketplace [26]. These practices shape Black consumers’ food environments and food choices and may contribute to poor nutrition and nutrition-related disparities [26,27].

Most food marketing studies focus on children due to their inability to recognize persuasive intent [13] and the oftentimes stronger political will to protect children through food marketing regulations [30]. Young adults (defined herein as those aged 18-24 years) may also be vulnerable for several reasons. They experience greater independence from childhood support systems and increasing agency and expectations of self-sufficiency, including obtaining and cooking their own food [31,32]. While young adults are beginning to accumulate social capital, their disposable incomes may still be less than those of older adults [31,32]. Young adults also have poorer diets [33,34] and are at increased risk of excessive weight gain [35]. Evidence also suggests that inhibitory control continues to mature into early adulthood [36]. There is an increasing awareness of food and nutrition insecurity during young adulthood [37-39], which may increase the appeal of advertisements that market low-cost, unhealthy foods [40]. Finally, young adults are a primary target of unhealthy food marketing in this new digital era [25].

Data on the effects of food marketing exposure among young adults are mixed [41,42]. In one study [41] that examined exposure to television-based food marketing in a sample of 82 young adults (average age 20.6 years), women who were exposed to two food commercials, each lasting 3 minutes, embedded within a 30-minute nature film, consumed significantly more snacks than women exposed to the neutral condition (approximately 66 g). The authors found the opposite pattern among men [41]. Another study [42] examined the effect of exposure to unhealthy versus healthy sponsored food commercials in an online sample of 1132 Australian young adults (aged 18-24 years). Young adults exposed to unhealthy sponsored food commercials showed greater brand awareness and more favorable attitudes toward branded items, but there was no effect on preferences for unhealthy foods [42]. In another study [8] that examined the effect of exposure to food advertisements across multiple platforms using ecological momentary assessment, young adults reported feeling hungrier and having more cravings when assessed after exposure to food advertisements compared with other assessments. Young adult samples are mainly convenience samples from colleges and universities, which limits generalizability [41,42]. Most experimental studies focus primarily on television advertisements [43]. No study has examined the effect of unhealthy food advertisement exposure on Black young adults (including those outside of college and university settings) who may be vulnerable to newer forms of digital-based food and beverage marketing. Additionally, no study has yet examined virtual reality (VR) as an emerging technology platform for unhealthy food and beverage marketing [44].

### VR-Based Fast-Food Marketing

Food and beverage companies are now harnessing advanced technologies to create immersive VR experiences. VR uses artificial intelligence and machine learning to psychologically immerse individuals in a computer-simulated and lifelike environment [45]. According to market reports, as of 2020, approximately 20% of US adults (more than 66 million) reported ever or currently using VR—a 16% increase from 2019 [46]. VR use is most popular among young people, as nearly two-thirds of all VR users identify as youth or young adults (34% of users reported being aged 16-23 years, 35% reported being aged 24-34 years) [47]. To access the most immersive forms of VR, users wear specialized headsets that engulf the entire field of view while presenting continually updated visual stimuli based on their movements and selections [48]. The unique elements of VR—which include *immersion,* engendering a sense of *presence*, and helping users develop a sense of *embodiment* with their digital selves—combine with traditional food and beverage marketing techniques that are designed to elicit biological responses that trigger a motivation to purchase and consume foods [49].

### Aim/Hypothesis 1

Exposure to food marketing contributes to stronger preferences for advertised foods [3-6] and higher purchase intention [7], and young adults may be particularly vulnerable to the effects of digital-based food and beverage marketing. Thus, we hypothesize that exposure to VR-based fast-food marketing will lead to higher purchase intention compared with the VR nonfood control in our sample of young adults. Due to marketing practices targeting Black communities, Black young adults may be especially vulnerable to the effects of food marketing exposure [24,25]. We hypothesize that Black young adults (compared with White young adults) will demonstrate a higher fast-food purchase intention.

### Motivations to Purchase: Arousal and Hunger

VR-based food and beverage marketing may more powerfully affect motivations to purchase and consume foods [50]. This study will examine 2 biological signal activations via: (1) electrodermal activity and (2) salivation. Electrodermal activity, measured through skin conductance (also referred to as galvanic skin response), is one of the most common ways in which food marketers examine marketing effectiveness [51]. Electrodermal activity refers to the changes in the electrical properties of the skin due to sweat secretion and has been closely linked to autonomic, emotional, and cognitive processing and sympathetic activity [52]. Electrodermal activity includes 2 types of electrical conductivity measurements in the skin: tonic (general changes in autonomic arousal measured by skin conductance level) and phasic (rapid skin conductance response [SCR] in relation to specific stimuli). SCR measures the electrical conductance of skin due to sweat secretion from the eccrine glands, which are involved in emotion-evoked sweating and are most dense in the palms and soles of the feet. With more activation (ie, arousal), there will be more eccrine sweat secretion and a larger SCR [53]. SCR can provide information on responses to brand preferences [54,55] and consumer behavior [56]. Although data in adults are mixed [51,57], in a study examining SCR in children (aged 8-11 years), those who viewed images of their favorite food or beverage brands showed a larger SCR than those who viewed unbranded foods or beverages [56].

Salivation—one of the most researched signals within food cue research—has been linked to the desire to consume foods, although data are mixed [58-63]. The release of saliva in the oral cavity, known as the cephalic-phase salivary response, supports chewing, swallowing, and digestion [58]. On the basis of the cue reactivity model, food cues from food advertisements may subconsciously activate biological reactions that affect decision-making and precipitate eating [64-67]. Multisensory food cue exposure—which occurs in VR experiences—may lead to more profound salivary effects and, subsequently, a desire to consume foods [67]. In a sample exposed to 12 conditions of increasing food cue sensory exposure, higher exposure led to greater salivation secretion [68]. In another examination of the effect of food marketing exposure on total salivation secretion and food intake in adult women (aged 20-64 years) of varying weight status [49], salivation significantly increased following exposure to a food commercial among women with overweight. A multisensory VR-based food and beverage marketing exposure may elicit an even stronger biological response than television. The authors did not measure whether the effect was stronger in younger adults (who may be more susceptible to food marketing) compared with older adults [49].

### Aims/Hypotheses 2 and 3

VR-based food and beverage marketing may more powerfully affect biological signals, prompting motivations to purchase and consume foods [50]. On the basis of data on children, exposure to unhealthy food marketing may lead to stronger arousal as measured by electrodermal amplitude or SCR [56]. Similarly, women with overweight have demonstrated increased salivation following exposure to unhealthy food marketing [49]. No study has combined multiple biological signals (electrodermal activity or SCR and salivation) or examined whether exposure to VR food marketing affects purchase intention among Black young adults [69]. We hypothesize that larger electrodermal amplitude, SCR, and salivary reactivity will be associated with higher fast-food purchase intention in those randomized to VR fast-food marketing compared with the control group. As previously noted, young adults may be particularly susceptible to food and nutrition insecurity [37-39], which may increase the appeal of unhealthy food advertisements [40]. Targeting Black communities may also exploit economic hardships, especially among those with food or nutrition insecurity [26]. To explore the interactions among race, ethnicity, food and nutrition insecurity, biological signal activation, and purchase intention among Black young adults exposed to VR fast-food marketing, we pose the following research question: Are food and nutrition insecurity, stronger electrodermal amplitude, and higher salivary reactivity associated with higher fast-food purchase intention among Black young adults randomized to VR fast-food marketing compared with those randomized to the control condition?

### Review of the Aims and Hypotheses

The goal of this manuscript is to describe a novel study protocol to examine the effects of VR-based fast-food marketing (compared to a VR-based nonfood control; hereafter referred to as “VR fast-food” and “VR nonfood”) on purchase intention, arousal, and hunger signals in a sample of Black and White young adults. Our aims and hypotheses are discussed subsequently.

### Aims and Hypotheses

The aims and hypotheses of this study are presented in Textbox 1.

**Aims and hypotheses.**

**Aim 1: determine the extent to which virtual reality (VR) fast-food marketing influences purchase intention and whether the effects are more pronounced in Black young adults.**

- Hypothesis 1.1: exposure to VR fast-food marketing will influence purchase intention compared with the VR nonfood control.
- Hypothesis 1.2: Black (compared with White) young adults exposed to VR fast-food marketing will demonstrate higher fast-food purchase intention.

**Aim 2: understand the influence of biological signal activation on the relationship between VR fast-food marketing and fast-food purchase intention.**

- Hypothesis 2.1: a larger electrodermal amplitude will be associated with higher fast-food purchase intention in those randomized to VR fast-food marketing compared with the control.
- Hypothesis 2.2: higher salivary reactivity will be associated with higher fast-food purchase intention in those randomized to VR fast-food marketing compared with the control.

**Aim 3 (exploratory): explore the interactions among race, ethnicity, food and nutrition insecurity, biological signal activation, and purchase intention among Black young adults exposed to VR fast-food marketing.**

- Hypothesis 3.1: food and nutrition insecurity along with stronger electrodermal amplitude are associated with higher fast-food purchase intention among Black young adults randomized to VR fast-food marketing exposure compared to those randomized to the control condition?
- Hypothesis 3.2: food and nutrition insecurity along with higher salivary reactivity are associated with higher fast-food purchase intention among Black young adults randomized to VR fast-food marketing exposure compared to those randomized to the control condition?

### Conceptual Framework

**Figure 1:**
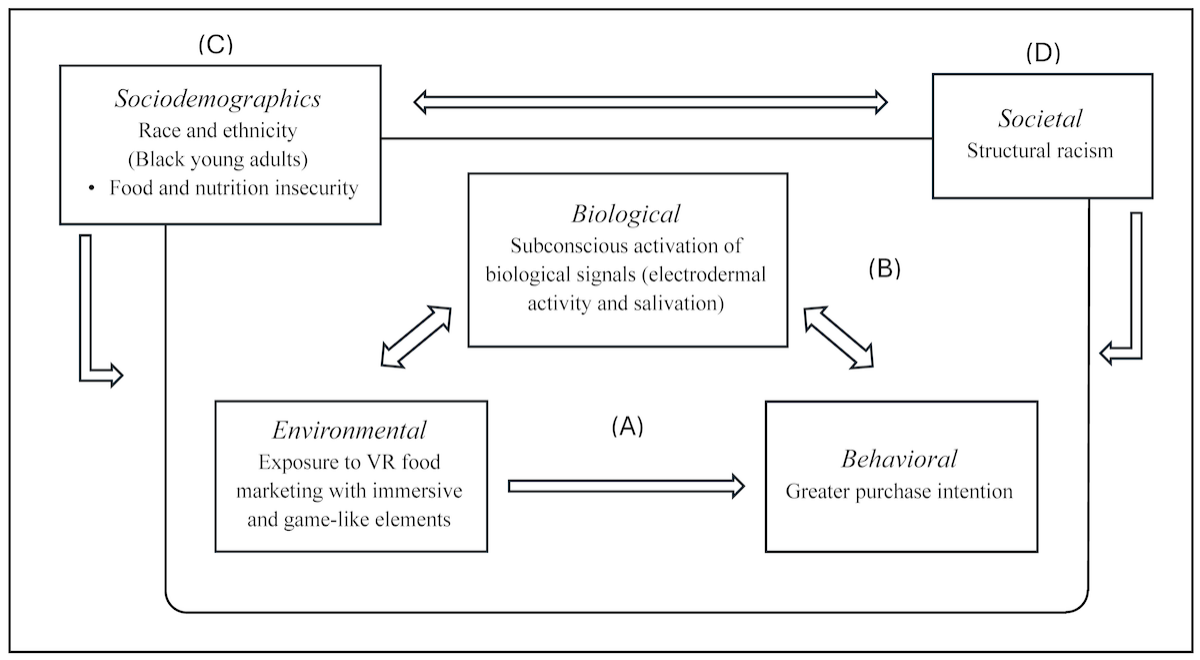
Conceptual framework. Illustrating the interactions among sociodemographic, societal, environmental, biological, and behavioral factors.

The study will be guided by a conceptual framework (Figure 1) based on evidence-based theories of food and beverage cues in the digital environment [50], theories of food cue reactivity [64-67], and the influence of structural racism on unhealthy food and beverage marketing [26]. (1) While young people experience immersion, presence, and embodiment within the VR experience—key enhancing elements unique to digital marketing [50]—a number of food cues that are presented throughout the experience are below conscious awareness. Exposure to food and beverage marketing influences preference, purchase intention, and consumption in young people [7,70]. (2) On the basis of the cue reactivity model, such cues may subconsciously activate biological responses (electrodermal activity and salivation) that can affect decision-making and purchase intention [64-67]. (3) Black young adults, particularly those with food and nutrition insecurity, may be a subset who are highly targeted [24,25], for whom racialized marketing on digital platforms may hold particular salience [26], and who may be especially vulnerable to poor diets [33,71]. (4) Black young adults may also be uniquely vulnerable to this process because of the influential role of structural racism [27]. According to the racialized marketing of unhealthy foods and beverages theory [26], structural racism (eg, racist structures, lack of political power, and income and wealth inequalities) functions in the background and leads to disproportionate exposure to unhealthy food and beverage marketing. Such exposure may then be more effective due to the: (1) normalization of unhealthy food and beverage consumption among Black consumers, (2) racial and ethnic tailoring that increases the salience of marketing techniques, (3) exploitation of price sensitivity due to income inequities in many Black communities, and (4) greater trust and responsiveness to marketing, especially after a history of being excluded from marketing [26].

## Methods

### Study Design

**Figure 2:**
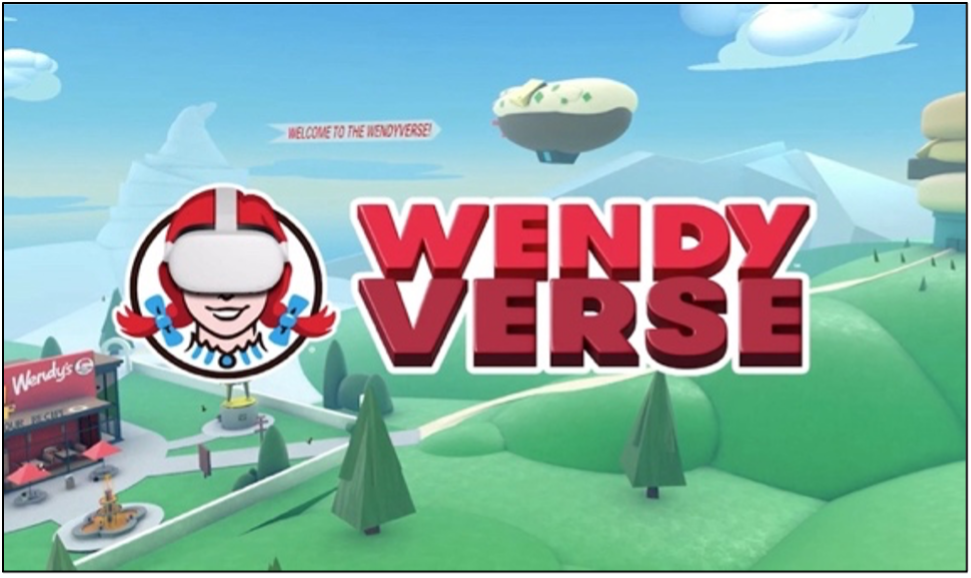
Screen capture of the home screen for the virtual reality (VR)–based fast-food marketing experience (experimental condition). The figure shows the Wendyverse VR app, where users can order from a restaurant operated by Wendy’s, play games, meet with others who may be visiting the Wendyverse, and access codes that can be used to obtain free food at physical restaurants. The VR app is commercially available [72] (as of March 25, 2025).

**Figure 3:**
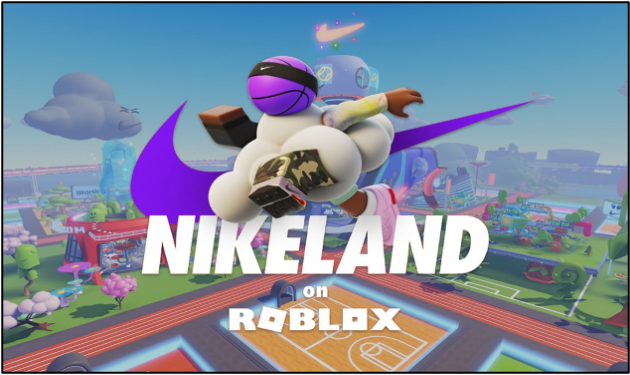
Screen capture of the home screen for the virtual reality (VR) nonfood experience (control condition). The figure shows the Nikeland VR app, where users can play sports, try on apparel, and engage with celebrity athletes. The VR app is commercially available [75] (as of March 25, 2025).

We will use a 2 x 2 randomized controlled experimental factorial design to examine the effect of VR fast-food marketing (compared to a VR nonfood control) on purchase intention, arousal, and hunger signals in a sample of Black and White young adults. The principal investigator (PI) and study team members will be approved to assign participants to the experimental and control conditions based on a 1:1 number generator managed by the PI. Participants will be randomized to either the VR food marketing condition (“Wendyverse”) or the VR nonfood control. Participants will be blinded to their condition until the end of the study (refer to the Secondary Measurements section). The Wendyverse (Figure 2 [72]) was created in 2022 by Wendy’s and is the first fast-food branded stand-alone VR world [73,74]. In the Wendyverse, users can order from a restaurant operated by Wendy’s, play games, meet with others who may be visiting the Wendyverse, and access codes that can be used to obtain free food at physical restaurants. The control condition will be the Nikeland app (Nike, Inc). In Nikeland (Figure 3 [75]), participants can play sports, try on apparel, and engage with celebrity athletes [76]. Researchers will ensure that the VR experiences are matched on engagement and that the control condition does not include any references to food or food and beverage brands. The primary outcomes will be purchase intention (assessed via self-report questionnaire), arousal (assessed via electrodermal activity), and hunger (assessed via salivary reactivity). We will also collect sociodemographic data (eg, income) and behavioral data (eg, media exposure) via questionnaires. On the basis of previous food marketing experimental research standards, the primary research objective of the study—to assess the effect of VR fast-food marketing exposure—will be concealed from participants until after they complete all study procedures. The true purpose of the study will be revealed during a poststudy debrief [77]. This method is intended to ensure that participants’ responses are not affected by performance biases or demand characteristics. Concealment is widely used in food marketing research and has been successfully used by the PI (OC) and the study team [77]. We used the SPIRIT checklist when writing our report Multimedia Appendix 1 [78]. This study is registered at ClinicalTrials.gov (NCT06917391) Multimedia Appendix 2.

### Participants and Recruitment

The study will enroll a sample of 100 (50%) Black and 100 (50%) White young adults from the New York City metropolitan area for a total of 200 participants (n=50, 25% pilot participants). The inclusion and exclusion criteria are provided in Textbox 2. The participants will be recruited through direct mailings, flyers, social media, listservs, and engagement with community organizations and a community advisory board. The study materials will invite the participants who are interested in sharing their perspectives on how young adults engage with and experience VR as a new technology.

**Inclusion and exclusion criteria.**

**Inclusion criteria**

- Aged 18-24 years
- Self-identify as a Black or White individual
- Self-report normal or corrected-to-normal vision from glasses or contacts
- Self-report comfort and ability to walk around within a VR paradigm
- Able to read, write, understand, and respond to all study materials (including the VR paradigm) in English
- Residing in the New York City metropolitan area
- Capacity and willingness to provide consent

**Exclusion criteria**

- Report a history of disorders causing motion sickness or have a history of significant motion sickness, which could be triggered within the VR paradigm
- Self-report dietary restriction, such as food allergies and veganism, which may lead to different effects when exposed to food marketing
- Self-report disliking ≥50% or more of the snacks that will be offered during the laboratory test snack as determined by a score of a score of ≤5 on the food preferences questionnaire, which may have a confounding effect on consumption
- Report being pregnant, breastfeeding, which may affect appetite
- Smoke or consume tobacco, which decreases salivary flow
- Self-report being very hungry based on 100-mm VAS hunger rating, which minimizes the burden to participants to fast prior to the visit and maximizes the ability to detect differences between groups. Those excluded for being very hungry will have the option of returning to the visit on a different day.

### Ethical Considerations

#### Ethics Approval and Informed Consent

The study was approved by the institutional review boards of the NYU Grossman School of Medicine and NYU Langone Health (i24-00910) in October 2024. Consent forms describing, in detail, the study, study procedures, and risks (Multimedia Appendix 3) will be sent to eligible participants after they complete the telephone prescreen and before the in-person study visit.

Upon arrival for the study visit, all participants will receive a verbal explanation, in terms suited to their comprehension, of the purposes, procedures, and potential risks of the study and of their rights as research participants. The participants will have the opportunity to carefully review the written consent form and ask questions before signing. The participants will also have the opportunity to discuss the study with their families or consider it before agreeing to participate. To ensure that biases do not affect outcomes, the primary purpose of the study will be concealed until participants complete all study components. At the end of the study or when a participant withdraws, we will disclose the true purpose of the study, solicit questions, and, for participants who have completed the entire study, conduct a second consent procedure after disclosing that the purpose was to assess the effects of VR food marketing exposure on the outcomes.

Each participant will sign the informed consent document before any procedures are conducted specifically for the study. The participants may withdraw consent at any time throughout the course of the study. If a participant withdraws, any information already collected and shared will be retained and analyzed, with the participants’ permission. The study team will provide participants with information on how to send a written notice (via email) to the study team with their request to withdraw. If a participant withdraws, no additional data will be collected. If a participant is scheduled for a visit but does not show (“no show”), no more than 3 attempts will be made to contact and reschedule. After that point, the study team will withdraw the participant from the study. If the participant later expresses interest in participating in the study, the study team will rescreen them for eligibility and schedule a visit. If the participant “no shows” again, another 3 attempts will be made to contact and reschedule. After that point, the study team will withdraw the participant from the study, and they will not be able to participate in the study in the future.

A copy of the signed informed consent will be given to each participant for their records. The rights and welfare of the participants will be protected by emphasizing that the quality of their medical care or their ability to receive any medical care at the institution will not be prohibited while participating in the study, nor will it be adversely affected if they decline to participate in the study.

#### Privacy and Confidentiality

Participant confidentiality will be strictly held in trust by the participating investigators and the study team. Therefore, the study protocol, documentation, data, and all other information generated will be held in strict confidence. No information concerning the study or the data will be released to any unauthorized third party without previous written consent or approval by the participant. Each participant’s contact information will be securely stored during the study. At the end of the study, all records will continue to be kept in a secure location for as long a period as dictated by the local institutional review board and institutional regulations. The research data of the study participants, which are collected for purposes of statistical analysis and scientific reporting, will be transmitted to and stored in an institutionally managed network shared drive set up for the study. The study data will not include the participant’s contact or identifying information. Rather, individual participants and their research data will be identified by a unique participant identification number. At the end of the study, the study databases will be deidentified and archived.

#### Compensation

The participants will receive a US $50 gift card for participating in the study and will be reimbursed for travel. If a participant does not complete the entire study, withdraws, or if the study is prematurely terminated, the participant’s payment will be prorated based on the portion of the study completed, including travel costs. All compensation plans will adhere to institutional policies on human participant payments.

### Procedure

After interested individuals contact the study team, a telephone screen will be conducted to determine their preliminary eligibility. Participants who may be eligible will be scheduled for a 2-hour in-person laboratory visit at the NYU Langone Health. Before arrival, the participants will be given instructions to prepare for the study visit, including eating before the visit (per the hunger exclusion criteria) and wearing comfortable clothing. The participants will also be given a copy of the consent form to review before the study visit. Once the participants arrive, the purpose, procedures, risks, and benefits of the study will be explained to them before they sign the consent form. One minimal risk of VR is cybersickness (motion sickness due to VR) [80]. The risk is significantly reduced by excluding individuals with a high propensity toward motion sickness, limiting exposure to 15 minutes [80], and using modern VR systems that reduce time delays between VR movements (also known as “lag time”) [79]. Potential participants will be informed of their right to withdraw from the protocol at any point, including if they experience any physical discomfort or cybersickness. After consenting to participate in the study, the participants will complete hunger ratings [81]. Those who report being “very hungry” before VR exposure will be rescheduled for a different visit.

### Experiment

The participants will receive a 5-minute tutorial on using the VR equipment [82]. The study team will place the VR headset (Meta Quest Pro) onto the participants’ heads, engulfing their field of view. The participants will practice walking and making selections in a neutral VR experience that will differ from the experimental conditions. After ensuring that the participants are comfortable within the VR environment, they will be given 15 minutes to explore and provide feedback on whether they believe their peers would engage with the experience (as part of the concealment procedure).

### Measures

#### Purchase Intention

After the exposure, the participants will complete a widely used measure assessing their likelihood of purchasing food from Wendy’s or any fast-food restaurant [83].

#### Electrodermal Activity and SCR

The EDA100C (Biopac Systems, Inc) will be used to measure the participants’ SCR throughout the VR exposure and will be integrated into the VR system. Two Ag-AgCl electrodes (1080FG, UFI) will be taped to the distal phalanx of the index and ring fingers on each participant’s nondominant hand. A small amount of biopotential contact medium gel (GEL101, Biopac Systems, Inc) will be applied to the electrodes, and a constant voltage of 0.5 V will be applied across the electrode pair. The experiment will be conducted in a suitable environment for the measurement (eg, temperature of approximately 71-75 F, 22 °C -24 °C) [52].

#### Salivation

A 3.5-cm dental roll will be placed horizontally under the participants’ tongues for 30 seconds before and after VR exposure. Salivation will not be collected during the exposure so as not to disrupt immersion. This noninvasive and sensitive measurement has been widely used [49,58-63].

### Secondary Measurements

Sociodemographic data, media exposure, and anthropometrics will also be measured. Food and nutrition insecurity will be measured using the 18-item US Household Food Security Survey [84]. We will conduct a semistructured postexperience interview and debrief to determine whether the concealment was successful. On the basis of previous food marketing experimental research standards, the primary research objective of the study—to assess the effect of food marketing exposure—will be concealed from the participants until after the study is completed during the poststudy interview and debrief. During the poststudy interview, we will ask participants the following questions: (1) How was your participation in the study? (warm-up), (2) What did you like most about participating in this study? (warm-up), (3) What did you like least about participating in this study? (warm-up), and (4) In your own words, what was this study about? (open-ended to allow the participants to share their understanding of the true purpose of the study). Following the interview, a study team member will respond to the following question: Was the concealment successful? If a participant did not guess the true purpose of the study, the response will be “yes” (the concealment was successful). If a participant correctly guessed the true purpose of the study, the response will be “no” (the concealment was not successful). After the debrief, the participants will be given verbal and written information about the true purpose of the study. The participants will be asked to reconsent after the true purpose of the study is explained.

### Analytic Plan

#### Sample Size Estimation

The sample size and effect size estimations are based on previous studies, power analyses, and calculations aligned with the primary objective of examining the effect of VR food marketing on purchase intention. In experimental food marketing studies in youth and adults [12,14], sample sizes have ranged from 92 to 118 participants (Cohen *d*=0.35-0.96; Cohen *f*^2^=0.06-0.15). In studies examining biological measures [49,56], sample sizes have ranged from 48 to 55 participants (Cohen *d*=0.4). After accounting for the relevant covariates in the model and for retention, to detect a medium effect size (Cohen *f*=0.25) with a 2-sided *α*=.05 and 80% power, an a priori analysis indicated that at least 128 participants would need to be randomized.

#### Statistical Analyses

To determine the extent to which VR fast-food marketing influences purchase intention, we will use a 1-way analysis of covariance to test the hypothesis that exposure to VR fast-food marketing influences purchase intention (ie, hypothesis 1.1). VR marketing (fast-food vs nonfood) will be entered as the independent variable, and self-reported purchase intention as the dependent variable. To examine whether the relationship is more pronounced in Black young adults (ie, hypothesis 1.2), a 2-way analysis of covariance will be used, with VR marketing (fast-food vs nonfood) and race and ethnicity (Black young adults vs White young adults) entered as independent variables and self-reported purchase intention as the dependent variable. To understand the influence of biological signal activation on the relationship between VR food marketing and purchase intention, we will use a multiple linear regression to test the hypothesis that electrodermal amplitude will moderate the relationship between VR food marketing and purchase intention (ie, hypothesis 2.1). VR marketing (fast-food vs nonfood), electrodermal amplitude, and their interaction terms will be included as predictors in the model. Multiple linear regressions will also be used to test the hypothesis that elevated salivary reactivity will moderate the relationship between VR food marketing and purchase intention (ie, hypothesis 2.2). This analysis will involve VR marketing (fast-food vs nonfood), salivary reactivity, and their interaction terms as separate predictors. A positive coefficient would indicate that the effect of VR marketing on purchase intention is stronger for individuals with higher levels of salivary reactivity (or electrodermal activity) than for those with lower levels of salivary reactivity (or electrodermal activity).

To explore the interactions among race and ethnicity, food and nutrition insecurity, biological signal activation, and purchase intention among Black young adults exposed to VR fast-food marketing (compared with the VR nonfood control), multiple linear regression analyses will be used to examine the hypothesis that food insecurity and stronger electrodermal amplitude correlate with greater fast-food purchase intention (ie, hypothesis 3.1). Food insecurity, salivary reactivity, their interaction terms, and an indicator of VR marketing exposure will be entered separately as predictors in the model. Similarly, multiple linear regressions will be used to test whether food insecurity and higher salivary reactivity are associated with stronger fast-food purchase intention (ie, hypothesis 3.2). Food insecurity, electrodermal amplitude, their interaction terms, and an indicator of VR marketing exposure will be entered separately as predictors. We will also explore income and education as individual-level socioeconomic status (SES) proxies. For each analysis, potential covariates will be considered, including race, sex, weight status, hunger, food insecurity, SES, study date and time, media use, and state mood. Covariates that are significantly associated with the outcomes will remain in the model. All data will be screened for missingness, outliers, and normality. To determine the best statistical method to handle missing data (eg, pairwise deletion or mean imputation), we will identify the type of missing data, percentage of missingness, data distribution, and impact on analyses. To minimize influences on analyses, we will consider recoding extreme outliers to 1.5 times the IQR below the 25th percentile or above the 75th percentile [70]. If biological data are nonnormally distributed, we will consider transformations or analyses that are suited to nonnormal data.

## Results

**Figure 4:**
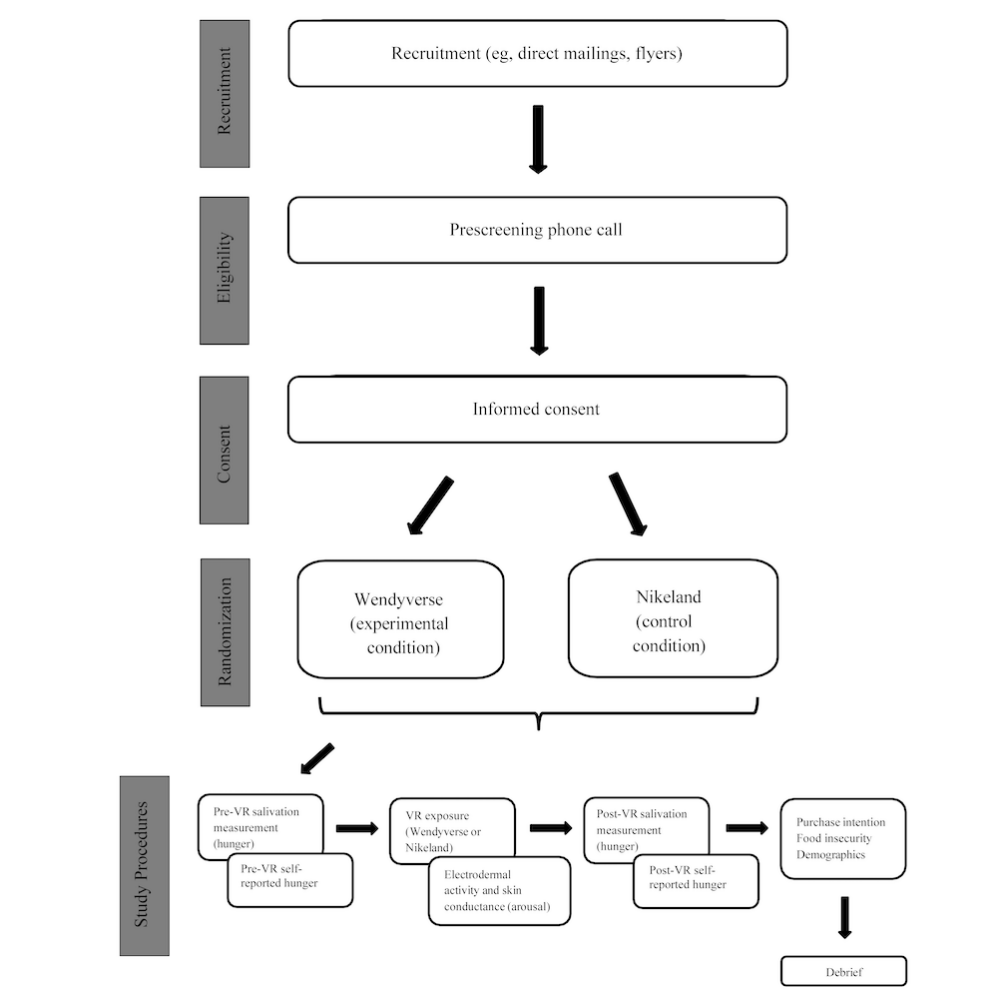
Study flow.

The study protocol was funded by the National Institute on Minority Health and Health Disparities in September 2024 (1K01MD019320). Recruitment is expected to begin in September 2025. The study involvement includes a 1-time, 2-hour laboratory visit (for the study flow, see Figure 4). We expect to complete data collection by October 2026. We will begin data cleaning and analysis in November 2026. Manuscript preparation is expected to begin in December 2026 and continue through March 2028.

## Discussion

### Anticipated Findings

Exposure to unhealthy food and beverage marketing is a public health concern [30]. Although most studies have examined television-based food and beverage marketing and recent studies have started exploring social media and gaming [85-87], VR food and beverage marketing has yet to be explored [44]. Young adults may be especially vulnerable to newer forms of food and beverage marketing on digital platforms, such as VR. The objective of this study is to explore the effect of VR-based fast-food marketing (compared to a VR nonfood control) on purchase intention, arousal, and hunger in a sample of Black and White young adults. We anticipate that young adults exposed to VR-based fast-food marketing will demonstrate greater purchase intention, higher arousal, and greater hunger than those in the VR nonfood control. Due to disproportionate exposure among Black communities, Black young adults may be especially vulnerable to the effects of unhealthy food and beverage marketing. Furthermore, unhealthy food and beverage advertisements may be uniquely appealing to young adults with food and nutrition insecurity. Thus, we also anticipate differences in primary outcomes based on race and ethnicity, as well as food and nutrition insecurity.

Although previous data consistently demonstrate the negative effects of exposure to unhealthy food and beverage marketing on children’s eating-related outcomes, data on the effects of food marketing exposure among young adults are mixed [41,42]. If hypothesis 1.1 is supported and the results show that young adults exposed to the VR-based fast-food marketing condition demonstrate greater purchase intention than those in the VR nonfood control, it would support the conceptualization that young adults may be susceptible to digital-based food and beverage marketing. While data examining similar outcomes have been mixed, such discrepancy may be due, in part, to previous sampling methods focused mainly on convenience samples in university or college settings and study designs examining television-based advertisements [41,42]. This study will improve upon these limitations by recruiting a sample of Black and White young adults from the community and exploring VR as a newer form of technology that is more appealing to young adults [44]. Research has also consistently shown that Black and lower-income communities are disproportionately exposed to more food advertisements and to the least healthy food advertisements than their counterparts [1,28,29]. However, this study will be the first to examine the unique effects of exposure on purchase intention, arousal, and hunger among a sample of Black young adults. Such data will be critical to a better understanding of how digital-based unhealthy food and beverage marketing may contribute to a poor diet among Black communities. Finally, our study will be the first to examine how race and ethnicity interact with food and nutrition security (a proxy for SES) [26,37-40] to exacerbate the negative effects of exposure to digital forms of unhealthy food and beverage marketing in young adults.

### Strengths and Limitations

This research has several strengths. As of March 2025, this project will be the first food marketing study to use a VR paradigm—an emerging technology within food and beverage marketing. Food marketing has previously been examined as a single unit placed in a particular location (eg, television) for a specific amount of time (eg, 30 s). The VR paradigm represents a movement toward digital marketing that can be ubiquitously integrated across platforms and devices, without the usual bounds of space or time. Second, this will be the first study to examine the effect of VR food marketing exposure on multiple biological outcomes (electrodermal activity and SCR, and salivation) that may be affected more powerfully in a VR food marketing exposure. Third, this will be the first study to explore the interactional role of food and nutrition insecurity as a key socioeconomic condition that could affect responses to food and beverage marketing exposure.

There are also several limitations. This VR paradigm will only explore the effect of a single exposure to unhealthy food and beverage marketing. It is not feasible to capture the cumulative effects of repeated exposure to all food and beverage marketing in this protocol. However, previous research has demonstrated an effect using as few as 2 television advertisements. In the only meta-analysis examining the effect of food and beverage marketing duration on children’s food intake—the outcome most proximal to diet and potential weight gain—Russell et al [88] found that exposure to an average of 4.4 minutes of television advertisements and 5 minutes of advergames (advertisements embedded within games) led to a 60-kcal and 53-kcal increase in intake, respectively, compared with control conditions. This study will expose young adults to 15 minutes of a marketing experience, which is expected to demonstrate a measurable effect. It is not clear how potential effects would compare across other marketing platforms. It has been suggested that because of the immersive nature of VR marketing and sophisticated technologies powered by neuroscience, VR food and beverage marketing may more powerfully affect behaviors [71]. This project will provide preliminary evidence to support more adequately powered studies on the effects of exposures across different mediums, such as television and social media. We will use commercially available VR apps for this study. In the event that the Wendyverse app is discontinued, we will identify other accessible food and nonfood VR apps. If those apps also become unavailable, we will work with experienced collaborators to develop a VR experience with key elements from VR fast-food apps.

### Future Directions

Data will support future studies to investigate the interrelationships among race and ethnicity, food and nutrition insecurity, biological outcomes, and actual purchase and consumption when exposed to VR food marketing compared with other mediums (eg, social media). Examining the potential effects of VR food and beverage marketing while the technology is still in its infancy will generate the knowledge needed to prevent adverse health effects, particularly for vulnerable communities. These efforts will go a long way in supporting policies that address the full spectrum of digital food and beverage marketing to young people.

## Appendix

Multimedia Appendix 1 SPIRIT (Standard Protocol Items: Recommendations for Interventional Trials) checklist.

Multimedia Appendix 2 Updated World Health Organization trial registration dataset and other relevant details.

Multimedia Appendix 3 Sample consent form and related participant materials.

Multimedia Appendix 4 Peer review report by ZMD1 DRI (O1) - National Institute on Minority Health and Health Disparities Special Emphasis Panel (National Institutes of Health, USA).

Multimedia Appendix 5 CONSORT-eHEALTH checklist (V 1.6.1).

## Abbreviations

PI: principal investigator
SCR: skin conductance response
SES: socioeconomic status
VR: virtual reality

## Data Availability

Data sharing is not applicable to this article as no data sets were generated or analyzed during this study.
