# Supplementary material for: Examining the Effect of Virtual Reality–Based Fast-Food Marketing on Eating-Related Outcomes in Young Adults: Protocol for a Randomized Controlled Trial
Source: JMIR Res Protoc. 2025 Sep 22;14:e69096. doi: 10.2196/69096 (PMC12501532; doi:10.2196/69096)
Supplement: Multimedia Appendix 1 [file resprot_v14i1e69096_app1.pdf]

- Home
- Home

Checklist for reporting a protocol of a clinical trial

This checklist is relevant to protocols of clinical trials, and is based on the SPIRIT guidelines. Read more

Complete now

Or download and complete offline

- 0 / 51 items completed
- complete offline

Instructions

Complete this checklist by entering the page numbers from your manuscript where readers will find each of the items listed below.

Your article may not currently address all the items on the checklist. Please modify your text to include the missing information. If you are certain that an item does not apply, please write "n/a" and provide a short explanation.

Download your completed checklist and include it as an extra file when you submit to a journal.

Administrative information

1.

Title

Descriptive title identifying the study design, population, interventions, and, if applicable, trial acronym. Read more

1

2a

Trial registration

Trial identifier and registry name. If not yet registered, name of intended registry. Read more

27

2b

Trial registration: data set

All items from the World Health Organization Trial Registration Data Set. Read more

Appendix

3.

Protocol version

Date and version identifier. Read more

Appendix

4.

Funding

Sources and types of financial, material, and other support. Read more

24

5a

Roles and responsibilities: contributorship

Names, affiliations, and roles of protocol contributors. Read more

1, 26

5b

Roles and responsibilities: sponsor contact information

Name and contact information for the trial sponsor. Read more

Appendix

5c

Roles and responsibilities: sponsor and funder

Role of study sponsor and funders, if any, in study design; collection, management, analysis, and interpretation of data; writing of the report; and the decision to submit the report for publication, including whether they will have ultimate authority over any of these activities. Read more

26

5d

Roles and responsibilities: committees

Composition, roles, and responsibilities of the coordinating centre, steering committee, endpoint adjudication committee, data management team, and other individuals or groups overseeing the trial, if applicable (see Item 21a for data monitoring committee). Read more

n/a

Introduction

6a

Background and rationale

Description of research question and justification for undertaking the trial, including summary of relevant studies (published and unpublished) examining benefits and harms for each intervention. Read more

5-12

6b

Background and rationale: choice of comparators

Explanation for choice of comparators. Read more

12

7.

Objectives

Specific objectives or hypotheses. Read more

8, 9-11

8.

Trial design

Description of trial design including type of trial (eg, parallel group, crossover, factorial, single group), allocation ratio, and framework (eg, superiority, equivalence, non-inferiority, exploratory). Read more

12

Methods: Participants, interventions, and outcomes

9.

Study setting

Description of study settings (eg, community clinic, academic hospital) and list of countries where data will be collected. Reference to where list of study sites can be obtained. Read more

16

10.

Eligibility criteria

Inclusion and exclusion criteria for participants. If applicable, eligibility criteria for study centres and individuals who will perform the interventions (eg, surgeons, psychotherapists). Read more

Figure 2

11a

Interventions: description

Interventions for each group with sufficient detail to allow replication, including how and when they will be administered. Read more

16-17

11b

Interventions: modifications

Criteria for discontinuing or modifying allocated interventions for a given trial participant (eg, drug dose change in response to harms, participant request, or improving / worsening disease). Read more

Appendix

11c

Interventions: adherance

Strategies to improve adherence to intervention protocols, and any procedures for monitoring adherence (eg, drug tablet return; laboratory tests). Read more

Appendix

11d

Interventions: concomitant care

Relevant concomitant care and interventions that are permitted or prohibited during the trial. Read more

n/a

12.

Outcomes

Primary, secondary, and other outcomes, including the specific measurement variable (eg, systolic blood pressure), analysis metric (eg, change from baseline, final value, time to event), method of aggregation (eg, median, proportion), and time point for each outcome. Explanation of the clinical relevance of chosen efficacy and harm outcomes is strongly recommended. Read more

17-18, Appendix

13.

Participant timeline

Time schedule of enrolment, interventions (including any run-ins and washouts), assessments, and visits for participants. A schematic diagram is highly recommended (see Figure). Read more

Figure 2, Appendix

14.

Sample size

Estimated number of participants needed to achieve study objectives and how it was determined, including clinical and statistical assumptions supporting any sample size calculations. Read more

3, 13

15.

Recruitment

Strategies for achieving adequate participant enrolment to reach target sample size. Read more

13

Methods: Assignment of interventions (for controlled trials)

16a

Allocation: sequence generation

Method of generating the allocation sequence (eg, computer-generated random numbers), and list of any factors for stratification. To reduce predictability of a random sequence, details of any planned restriction (eg, blocking) should be provided in a separate document that is unavailable to those who enrol participants or assign interventions. Read more

12

16b

Allocation concealment mechanism

Mechanism of implementing the allocation sequence (eg, central telephone; sequentially numbered, opaque, sealed envelopes), describing any steps to conceal the sequence until interventions are assigned. Read more

12

16c

Allocation: implementation

Who will generate the allocation sequence, who will enrol participants, and who will assign participants to interventions. Read more

12

17a

Blinding (masking)

Who will be blinded after assignment to interventions (eg, trial participants, care providers, outcome assessors, data analysts), and how. Read more

12

17b

Blinding (masking): emergency unblinding

If blinded, circumstances under which unblinding is permissible, and procedure for revealing a participant's allocated intervention during the trial. Read more

n/a

Methods: Data collection, management, and analysis

18a

Data collection plan

Plans for assessment and collection of outcome, baseline, and other trial data, including any related processes to promote data quality (eg, duplicate measurements, training of assessors) and a description of study instruments (eg, questionnaires, laboratory tests) along with their reliability and validity, if known. Reference to where data collection forms can be found, if not in the protocol. Read more

19

18b

Data collection plan: retention

Plans to promote participant retention and complete follow-up, including list of any outcome data to be collected for participants who discontinue or deviate from intervention protocols. Read more

n/a

19.

Data management

Plans for data entry, coding, security, and storage, including any related processes to promote data quality (eg, double data entry; range checks for data values). Reference to where details of data management procedures can be found, if not in the protocol. Read more

Appendix

20a

Statistics: outcomes

Statistical methods for analysing primary and secondary outcomes. Reference to where other details of the statistical analysis plan can be found, if not in the protocol. Read more

19-20

20b

Statistics: additional analyses

Methods for any additional analyses (eg, subgroup and adjusted analyses). Read more

19-20

20c

Statistics: analysis population and missing data

Definition of analysis population relating to protocol non-adherence (eg, as randomised analysis), and any statistical methods to handle missing data (eg, multiple imputation). Read more

19-20

Methods: Monitoring

21a

Data monitoring: formal committee

Composition of data monitoring committee (DMC); summary of its role and reporting structure; statement of whether it is independent from the sponsor and competing interests; and reference to where further details about its charter can be found, if not in the protocol. Alternatively, an explanation of why a DMC is not needed. Read more

n/a

21b

Data monitoring: interim analysis

Description of any interim analyses and stopping guidelines, including who will have access to these interim results and make the final decision to terminate the trial. Read more

n/a

22.

Harms

Plans for collecting, assessing, reporting, and managing solicited and spontaneously reported adverse events and other unintended effects of trial interventions or trial conduct. Read more

Appendix

23.

Auditing

Frequency and procedures for auditing trial conduct, if any, and whether the process will be independent from investigators and the sponsor. Read more

n/a

Ethics and dissemination

24.

Research ethics approval

Plans for seeking research ethics committee / institutional review board (REC / IRB) approval. Read more

13

25.

Protocol amendments

Plans for communicating important protocol modifications (eg, changes to eligibility criteria, outcomes, analyses) to relevant parties (eg, investigators, REC / IRBs, trial participants, trial registries, journals, regulators). Read more

Appendix

26a

Consent or assent

Who will obtain informed consent or assent from potential trial participants or authorised surrogates, and how (see Item 32). Read more

13

26b

Consent or assent: ancillary studies

Additional consent provisions for collection and use of participant data and biological specimens in ancillary studies, if applicable. Read more

n/a

27.

Confidentiality

How personal information about potential and enrolled participants will be collected, shared, and maintained in order to protect confidentiality before, during, and after the trial. Read more

13, Appendix

28.

Declaration of interests

Financial and other competing interests for principal investigators for the overall trial and each study site. Read more

26

29.

Data access

Statement of who will have access to the final trial dataset, and disclosure of contractual agreements that limit such access for investigators. Read more

Appendix

30.

Ancillary and post trial care

Provisions, if any, for ancillary and post-trial care, and for compensation to those who suffer harm from trial participation. Read more

n/a

31a

Dissemination policy: trial results

Plans for investigators and sponsor to communicate trial results to participants, healthcare professionals, the public, and other relevant groups (eg, via publication, reporting in results databases, or other data sharing arrangements), including any publication restrictions. Read more

Appendix

31b

Dissemination policy: authorship

Authorship eligibility guidelines and any intended use of professional writers. Read more

Appendix

31c

Dissemination policy: reproducible research

Plans, if any, for granting public access to the full protocol, participant-level dataset, and statistical code. Read more

Appendix

Appendices

32.

Informed consent materials

Model consent form and other related documentation given to participants and authorised surrogates. Read more

Appendix

33.

Biological specimens

Plans for collection, laboratory evaluation, and storage of biological specimens for genetic or molecular analysis in the current trial and for future use in ancillary studies, if applicable. Read more

n/a

To acknowledge this checklist in your methods, please state "We used the SPIRIT checklist when writing our report [citation]". Then cite this checklist as Chan A-W, Tetzlaff JM, Gøtzsche PC, Altman DG, Mann H, Berlin J, Dickersin K, Hróbjartsson A, Schulz KF, Parulekar WR, Krleža-Jerić K, Laupacis A, Moher D. SPIRIT 2013 Explanation and Elaboration: Guidance for protocols of clinical trials. BMJ. 2013;346:e7586.

download completed checklist download

The SPIRIT Explanation and Elaboration paper is distributed under the terms of the Creative Commons Attribution License CC-BY-NC

- Top
- Complete offline
- Instructions
- Administrative information
- Introduction
- Methods: Participants, interventions, and outcomes
- Methods: Assignment of interventions (for controlled trials)
- Methods: Data collection, management, and analysis
- Methods: Monitoring
- Ethics and dissemination
- Appendices

Every clinical trial should be based on a protocol—a document that details the study rationale, proposed methods, organisation, and ethical considerations. Trial investigators and staff use protocols to document plans for study conduct at all stages from participant recruitment to results dissemination. Funding agencies, research ethics committees/institutional review boards, regulatory agencies, medical journals, systematic reviewers, and other groups rely on protocols to appraise the conduct and reporting of clinical trials.

Was this useful?

© 2020 Copyright EQUATOR Network, made in collaboration with penelope.ai

Privacy Policy Cookie
Update your advertising tracking preferences
