# Supplementary material for: Examining the Effect of Virtual Reality–Based Fast-Food Marketing on Eating-Related Outcomes in Young Adults: Protocol for a Randomized Controlled Trial
Source: JMIR Res Protoc. 2025 Sep 22;14:e69096. doi: 10.2196/69096 (PMC12501532; doi:10.2196/69096)
Supplement: Multimedia Appendix 2 [file resprot_v14i1e69096_app2.docx]

**Appendix 2**

**World Health Organization Trial Registration Data Set**

1. **Primary Registry and Trial Identifying Number**
   - ClinicalTrials.gov identifier: NCT06917391; https://clinicaltrials.gov/study/NCT06917391.
2. **Date of Registration in Primary Registry**
   - April 7, 2025
3. **Secondary Identifying Numbers**
   - National Institutes of Minority Health and Health Disparities, Unique Federal Award Identification Number (FAIN): K01MD019320
   - NYU Langone Health, IRB Protocol #i24-00910
4. **Source(s) of Monetary or Material Support**
   - National Institutes of Minority Health and Health Disparities, Grant # 1K01MD019320
5. **Primary Sponsor**
   - New York University School of Medicine
6. **Secondary Sponsor(s)**
   - None
7. **Contact for Public Queries**
   - Omni Cassidy, PhD, Address: 180 Madison Ave, 3^rd^ Floor, New York, NY, 10016; Email: [omni.cassidy@nyulangone.org](mailto:omni.cassidy@nyulangone.org); Phone: 646-501-3546
8. **Contact for Scientific Queries**
   - Omni Cassidy, PhD, Principal Investigator, Assistant Professor, NYU Grossman School of Medicine/Langone Health; Address: 180 Madison Ave, 3^rd^ Floor, New York, NY, 10016; Email: [omni.cassidy@nyulangone.org](mailto:omni.cassidy@nyulangone.org); Phone: 646-501-3546
9. **Public Title**
   - Examining the effect of virtual-reality based fast-food marketing on eating-related outcomes in young adults: Research Protocol for the Young Adults’ ExperienceS with Virtual Reality (YAES VR) Study
10. **Scientific Title**
    - The effect of virtual reality-based food marketing on biological hunger signals and purchase intention in young adults
11. **Countries of Recruitment**
    - United States of America
12. **Health Condition(s) or Problem(s) Studied**
    - Unhealthy food and beverage marketing; poor nutrition
13. **Intervention(s)**
    - The following conditions will be evaluated:
      - Experimental Condition: VR-based fast-food marketing (Wendyverse)
      - Experimental Description: VR app created by Wendy’s. In the Wendyverse, users can order from a Wendy’s restaurant, play games, meet with others who may be visiting the Wendyverse, and access codes that can be used to obtain free food at physical restaurants.
      - Control Condition: VR-based non-food control (Nikeland)
      - Control Description: VR app created by Nike, Inc. In Nikeland, participants can play sports, try on apparel, and engage with celebrity athletes. The control condition is matched on engagement and does not include any references to food or food/beverage brands.

1. **Key Inclusion and Exclusion Criteria**
   - Inclusion Criteria:
     1. 18-24 years old;
     2. self-identify as Black or white in order to determine racial/ethnic differences in responses to unhealthy VR-based food and beverage marketing;
     3. self-report normal or corrected-to-normal vision from glasses or contacts;
     4. self-report comfort and ability to walk around within a VR paradigm;
     5. able to read, write, understand, and respond to all study materials (including the VR paradigm) in English
     6. residing in the NYC-metropolitan area in order to be available for in-person study
     7. capacity and willingness to provide consent
   - Exclusion Criteria:
     1. report a history of disorders causing motion sickness or have a history of significant motion sickness, which could be triggered within the VR paradigm
     2. self-report dietary restriction, such as food allergies or veganism, which may lead to different effects when exposed to food marketing;
     3. Self-report disliking 50% or more of the snacks that will be offered during the laboratory test snack as determined by a score of 5 or less on the food preferences questionnaire, which may have a confounding effect on consumption;
     4. report being pregnant or breastfeeding, which may affect hunger;
     5. smoke or consume tobacco, which decreases salivary flow; or
     6. self-report being very hungry based on 100 mm VAS hunger rating, which minimizes the burden to participants to fast prior to the visit and maximizes the ability to detect differences between groups.100 Those excluded for being very hungry will have the option of returning to the visit on a different day.
2. **Study Type**
   Study type consists of:
   - Type of study: Observational
   - Study design including:
     - Method of allocation: Randomized
     - Masking: Participant only
     - Assignment: Factorial
     - Purpose: Participants will be randomized to examine the effect of exposure to VR-based fast-food marketing compared to a VR non-food control.
   - Phase (if applicable): N/A
3. **Date of First Enrollment**
   - April 2025 (anticipated)
4. **Sample Size**
   - N=200
   - Currently enrolled: None.
5. **Recruitment Status**
   - Recruitment status of this trial:
     - Pending: participants are not yet being recruited or enrolled at any site
6. **Primary Outcome(s)**
   - Outcome: Purchase intention

Metric/method of measurement: Self-report questionnaire

Timepoint: One-time, post VR exposure

Outcome: Salivary reactivity

Metric/method of measurement: Self-report questionnaire

Timepoint: Pre-VR exposure, post-VR exposure

Outcome: Electrodermal activity/skin conductance response

Metric/method of measurement: EDA100C device (Biopac Systems, Inc., Goleta, CA, USA)

Timepoint: Throughout VR exposure

1. **Key Secondary Outcomes**

- Outcome: Code selection

Metric/method of measurement: Self-report (yes/no)

Timepoint: Post-VR exposure

- Outcome: Food/nutrition insecurity

Metric/method of measurement: 18-item U.S. Household Food Security Survey

Timepoint: Post-VR exposure

1. **Ethics Review**
   - Status: Approved
   - Date of approval: October 6, 2024
   - Name and contact details of Ethics committee(s): NYU Langone Health, Phone: (212) 263-4110; Email: irb-info@nyulangone.org
2. **Completion date**
   - March 31, 2028 (anticipated)
3. **Summary Results**
   - Data/results have not yet been generated.
4. **IPD sharing statement**
   - Plan to share IPD: Yes
   - Plan description: Based on ethical and legal considerations, the data produced in the course of the project will be preserved and shared according to NIH Public Access Policy guidelines. All dataset(s) that can be shared will be deposited in the Open Science Framework and other relevant depositories (if any) for virtual reality, purchasing behavior, and salivation data among young adults. Data will be made available as soon as possible or at the time of associated publication. Data will be made available, at minimum, for seven years.

SPIRIT Guideline Checklist Items

**Protocol version**

October 2024, Version 1

**Name/Contact Information of Trial Sponsor**

New York University School of Medicine, 1 Park Ave, 6^th^ floor, New York, NY 10016

**Data monitoring**

The risks associated with this study are considered minimal and will be addressed in the protocol and consent form. It is the responsibility of the PI to oversee the study. This monitoring will include careful assessment and appropriate reporting of adverse events as noted above, as well as the construction and implementation of a data and safety-monitoring plan. Thus, an independent data monitoring committee is not currently indicated.

**Handling Adverse Events**

At each contact with the subject, the investigator will seek information on adverse events by specific questioning and, as appropriate, by examination. Information on all adverse events will be recorded immediately in the source document, and also in the appropriate adverse event module of the case report form (CRF).

All adverse events occurring during the study period will be recorded. The course of each event will be followed until resolution or until it has been determined that the study participation is not the cause. Serious adverse events that are still ongoing at the end of the study period will be followed up to determine the final outcome. Any serious adverse event that occurs after the study period and is considered to be possibly related to study participation will be recorded and reported immediately.

Reportable events noted above will be reported to the IRB using a Reportable New Information submission promptly, but no later than 5 working days, and will include a description of the event with information regarding its fulfillment of the above criteria, follow-up/resolution, and need for revision to consent form and/or other study documentation. Copies of each report and documentation of IRB notification and receipt will be kept in the PI’s study file.

**Improving Adherence/Recruitment and Retention Plan**

Young adults and individuals of color are historically challenging groups to recruit for research studies, partially due to historical misconduct within research and academic settings. To address this possibility, the PI and study team will work closely with consultants to establish a community health advisory board (CAB) comprised of 7-10 young adults representing the target study sample and ensuring recruitment is culturally-sensitive. Consultants includes individuals are part of the Community Engagement and Population Health Research core of NYU’s Clinical Translational Science Institute and the Community Engagement Pillar for the Institute for Excellence in Health Equity at NYU Grossman School of Medicine. These sections, along with the Department of Population Health (DPH)—the PI’s home department—includes a number of resources to support recruiting community participants, particularly communities that have been historically marginalized. Young adults recruited for the CAB will be compensated a total of $25 per hour for participating four times per year once established. The PI will identify a diverse group of adults who are representative of the target study sample based on race/ethnicity and gender, as well as other appropriate intersecting identities based on religious affiliation or sexual orientation. The PI will utilize culturally-appropriate strategies to support their participation (e.g., acknowledging the historical context of research participation). Young adults who participate in the study would be eligible to be participate only after they have completed the study. The CAB will assist in identifying groups, organizations, and employers from which the PI can recruit the remaining young adult study sample. The PI will also utilize my professional networks, which includes junior and senior researchers with extensive experience working with young people. The PI and study team will also attend workshops and trainings hosted by the NYU Langone Clinical and Translational Science Institute (CTSI) Integrating Special Populations Unit, which provides resources and trainings for conducting research with special populations, including underserved populations (e.g., racial and ethnic minorities) and those with health disparities.
