# Supplementary material for: Examining the Effect of Virtual Reality–Based Fast-Food Marketing on Eating-Related Outcomes in Young Adults: Protocol for a Randomized Controlled Trial
Source: JMIR Res Protoc. 2025 Sep 22;14:e69096. doi: 10.2196/69096 (PMC12501532; doi:10.2196/69096)
Supplement: Multimedia Appendix 3 [file resprot_v14i1e69096_app3.docx]

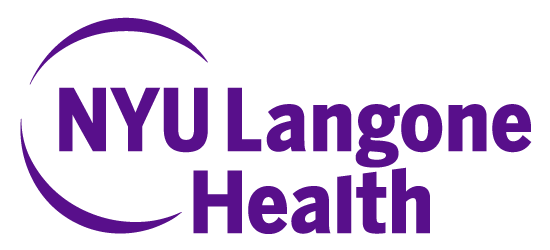
**Research Participant**

**Informed Consent Form**

| **Title of Study:** | Young Adults’ ExperienceS with Virtual Reality (YAES VR) Study |
| --- | --- |
| **Principal Investigator:** | Omni Cassidy, Ph.D.Department of Population Health NYU Langone Health  180 Madison Avenue, New York, NY 10016 Omni.Cassidy@nyulangone.org |
| **Emergency Contact:** | Omni Cassidy, Ph.D. Omni.Cassidy@nyulangone.org  646-501-3546 |

1. About volunteering for this research study

You are being invited to take part in a research study being conducted by Dr. Omni Cassidy, Department of Population Health, at NYU Langone Health (NYULH). Your participation is voluntary, which means you can choose whether or not you want to take part in this study.

People who agree to take part in research studies are called “subjects” or “research participants.” These words are used throughout this consent form. Before you can make your decision, you will need to know what the study is about, the possible risks and benefits of being in this study, and what you will have to do in this study. You may also decide to discuss this study and this form with your family or friends. If you have any questions about the study or this form, please ask us. If you decide to take part in this study, you must sign this form. We will give you a copy of this form signed by you for you to keep.

1. What is the purpose of this study?

The purpose of this research study is to understand more about young adults’ experiences and use of virtual reality (VR). In particular, we are interested in young adults’ feedback about new VR applications (“apps”). For scientific reasons, this consent form does not include all information about the research question being tested. The researchers will give you more information when your participation in the study is over.

1. How long will I be in the study? How many other people will be in the study?

Participation will involve a study visit at NYU Langone Health that will last about 2 hours. Up to 200 people will be in the study.

1. What will I be asked to do in the study?

If you agree to be in this study, we will ask you to sign this consent form first. We will schedule a study visit that will take place in person at 180 Madison Avenue at a time that is convenient for you. During the study visit, the following will take place:

- Like flipping a coin, you will be randomly assigned to play one of VR game apps. You will be asked to play the VR app while wearing VR headset that will go over your eyes. Before you play the VR app, we will give you a tutorial to make sure you feel comfortable using the controls and making selections in the VR app.
- You will be asked to give us a saliva sample from your mouth. We will provide you with a swab to put in your mouth for the saliva collection.
- While you are playing the VR app, we will also ask you to place a sensor on your hand that will give us information about how you may be feeling in the moment.
- We will give you pre-packaged snacks that you can eat.
- You will also be asked to complete some questionnaires that include questions about yourself including, but not limited to, your level of education, race/ethnicity, age, and others. You are free to skip any question you do not wish to answer.
- You will also have your body weight measured.

Any identifiable private information collected and/or used for the purposes of this research will not be used or distributed for future research studies.

1. What are the possible risks or discomforts?

**Risk of Study**

One possible physical risk is experiencing motion sickness while wearing the VR headset, which is referred to as cybersickness. To make sure this does not happen, people who get motion sick easily (because of an illness or other factors) are excluded from participating in this study. We also limit the amount of time you will use the VR app while wearing the headset to only 15 minutes, which will make it much less likely to feel cybersickness. If you feel physically uncomfortable, you can stop participating in the study. You may also feel bored or irritated with the survey questions.

Another possible risk is loss of confidentiality of your information. To make sure this does not happen, research records will be kept in a locked file; only the researchers will have access to the records.

1. What if new information becomes available?

During the course of this study, we may find more information that could be important to you. This includes information that might cause you to change your mind about being in the study. We will notify you as soon as possible if such information becomes available.

1. What are the possible benefits of the study?

You are not expected to get any benefit from being in this research study. The benefits to the scientific community would be a deeper understanding of the experiences young adults have with VR.

1. What other choices do I have if I do not participate?

Taking part in this study is completely voluntary. You have the option not to participate. Your decision will not affect your current or future relationship with NYU Langone Health.

1. Will I be paid for being in this study?

You will be paid with a $50 gift card at the end of the study for your participation and we will pay you back for travel costs to and from the study site. To be paid, you must give the receipts to the study staff. If you choose to leave or stop participating in the study for any reason before finishing the entire study*,* you will still be paid for the portion of the study you completed, and we will still pay you back for travel costs.

1. Will I have to pay for anything?

There are no costs to you as a participant for your participation in this study.

1. What happens if I am injured from being in the study?

For medical emergencies contact 911. If you think you have been injured as a result of taking part in this research study, tell the principal investigator or a study team member as soon as possible. The principal investigator’s name and phone number are listed at the top of page 1 of this consent form.

We will offer you the care needed to treat injuries directly resulting from taking part in this research. We may bill your insurance company or other third parties, if appropriate, for the costs of the care you get for the injury, but you may also be responsible for some of them.

There are no plans for the NYU Grossman School of Medicine or NYU Langone Health to pay you or give you other compensation for the injury. You do not give up your legal rights by signing this form.

1. When is the study over? Can I leave the Study before it ends?

The study will last about three years in total. If you decide to participate, you are free to leave the study at any time. Leaving the study will not interfere with your future care, payment for your health care or your eligibility for health care benefits.

1. How will you protect my confidentiality?

Your medical information is protected health information, or “PHI”, and is protected by federal and state laws, such as the Health Insurance Portability and Accountability Act, or HIPAA. This includes information in your research record as well as information in your medical record at NYU Langone Health. In compliance with NYU Langone Health policies and procedures and with HIPAA, only those individuals with a job purpose can access this information. Medical information created by this research study may become part of your medical record. We may include your research information in your medical record for several reasons, including for the billing of services provided in connection with the study, to securely document any medical services you receive, and so that other members of the NYU Langone Health community who may treat you have access to important information about your health.

You have a right to access information in your medical record. In some cases, when necessary to protect the integrity of the research, you will not be allowed to see or copy certain information relating to the study while the study is in progress, but you will have the right to see and copy the information once the study is over in accordance with NYU Langone Health policies and applicable law.

**Certificate of Confidentiality**

To help us further protect your confidentiality, this research is covered by a Certificate of Confidentiality from the National Institutes of Health (NIH). The NIH has issued a Certificate of Confidentiality for this research. This adds special protection for the research information (data, documents, or biospecimens) that may identify you.

Research information protected by this Certificate of Confidentiality cannot be disclosed to anyone else who is not connected with the research, without your consent. With this Certificate of Confidentiality, the researchers may not disclose or use research information that may identify you in any federal, state, or local civil, criminal, administrative, legislative, or other action, suit, or proceeding, or be used as evidence, for example, if there is a court subpoena, without your consent. However, disclosure, without your consent, is still necessary if there is a federal, state, or local law that requires disclosure (such as to report child abuse or communicable diseases).

The Certificate of Confidentiality cannot be used to refuse a request for information from appropriate government agencies responsible for project oversight.

The Certificate of Confidentiality does not prevent you from releasing information about yourself and your involvement in this research, including for your medical treatment. Federal regulations may also allow for the use or sharing of information for other scientific research.

By agreeing to be in this research and signing below, you are giving your consent to share research information with others at NYU Langone Health. This means that your research information, including lab results, X-rays, MRIs, information about the investigational drug used in this study, may be included in your NYU Langone Health electronic medical record.

1. HIPAA Authorization

As noted in the Confidentiality section above, federal law requires us, and our affiliated researchers, health care providers, and physician network to protect the privacy of information that identifies you and relates to your past, present, and future physical and mental health conditions. We are asking for your permission (authorization) to use and share your health information with others in connection with this study- in other words, for purposes of this research, including conducting and overseeing the study.

Your treatment outside of this study, payment for your health care, and your health care benefits will not be affected even if you do not authorize the use and disclosure of your information for this study.

**What information may be used or shared with others in connection with this study?**

All information in your research record for this study may be used and shared with those individuals listed in this section. You have a right to access information in your medical record. In some cases when necessary to protect the integrity of the research, you will not be allowed to see or copy certain information relating to the study while the study is in progress, but you will have the right to see and copy the information once the study is over in accordance with NYU Langone Health policies and applicable law.

**Who may use and share information in connection with this study?**

The following individuals may use, share or receive your information for this research study:

- - The Principal Investigator, study coordinators, other members of the research team, and personnel responsible for the support or oversight of the study.
  - The study sponsor: NIH
  - Governmental agencies responsible for research oversight (e.g., the Food and Drug Administration or FDA).

Your information may be re-disclosed or used for other purposes if the person who receives your information is not required by law to protect the privacy of the information.

**What if I do not want to give permission to use and share my information for this study?**

Signing this form is voluntary. You do not have to give us permission to use and share your information, but if you do not, you will not be able to participate in this study.

**Can I change my mind and withdraw permission to use or share my information?**

Yes, you may withdraw or take back your permission to use and share your health information at any time for this research study. If you withdraw your permission, we will not be able to take back information that has already been used or shared with others. To withdraw your permission, send a written notice to the principal investigator for the study noted at the top of page 1 of this form. If you withdraw your permission, you will not be able to stay in this study.

**How long may my information be used or shared?**

Your permission to use or share your personal health information for this study will never expire unless you withdraw it.

1. The Institutional Review Board (IRB) and how it protects you

The IRB reviews all human research studies – including this study. The IRB follows Federal Government rules and guidelines designed to protect the rights and welfare of the people taking part in the research studies. The IRB also reviews research to make sure the risks for all studies are as small as possible. The NYU Langone Health IRB Office number is 212-263-4110. The NYU Langone Health IRB is made up of doctors, nurses, non-scientists, and people from the Community.

1. Who can I call with questions, or if I’m concerned about my rights as a research participant?

If you have questions, concerns or complaints regarding your participation in this research study or if you have any questions about your rights as a research participant, you should speak with the Principal Investigator listed on top of the page 1 of this consent form. If a member of the research team cannot be reached or you want to talk to someone other than those working on the study, you may contact the NYU Langone Health IRB at 212-263-4110.

NYU Langone Health is committed to providing a safe, productive, and welcoming environment for participants and researchers in all research studies and interactions. All participants will be treated with respect and consideration, and in turn, we ask that you please treat fellow participants and research staff with respect. Please refer to [NYU Langone’s Statement on the Conduct of Participants in Research](https://med.nyu.edu/research/office-science-research/clinical-research/sites/default/files/nyulh-participant-statement-of-conduct.pdf) studies for further information.

1. Research with Applications, Software & Novel Technology

This study will use a VR headset (with built-in eye-tracking and integrated hand sensor) where you will see VR mobile applications (apps) to gather information for the researchers as part of this study. All VR apps will be downloaded and handled by the research study team. To provide the VR device, we are working with a company called WorldViz, which is a company that supports VR research. The VR headset is created by Meta and the VR apps are created by Meta Horizon Worlds and Roblox.

This VR headset is provided by the research study team for you to use on-site during the in-person study visit only. The VR headset and apps will be assigned to NYU Grossman School of Medicine and the study team. The vendors may retain data based on selections you make in the VR apps. However, your personal identity will not be connected to any of the selections; and the VR headset, apps, or vendors will not have any access to your personal information. If a username is required, the study team will use “YAES-VR” or a related username. To use the product or device, you must agree to the company’s rules before you can use it, just as if you bought the product or service for yourself. The researchers of the study do not control these rules. We will help you understand these rules in the “Terms of Service” or the “End User License Agreement” that come with the product or device. Please read these rules carefully. These rules may ask you to agree to certain things, like not to sue the company if something goes wrong with the product or device. These rules may also allow the company to get, keep, or give others a copy of your information that comes from the product or device. In this case, that may be the selections you make within the VR apps. Although this study will protect your personal information, we cannot protect or control what the company does with the copy that goes to them, such as your selections made in the VR app. If you do not agree to the company’s rules, you do not have to use the product or device. Taking part in this study is completely voluntary. You have the option not to participate. Your decision will not affect your current or future relationship with NYU Langone Health.

General Websites:

WorldViz: <https://www.worldviz.com/>

Meta: https://about.meta.com/

Roblox: https://corp.roblox.com/

Terms and Privacy Policies:

*We have provided links to the terms and privacy polices below. Some of this information may not apply to you. For example, you will not share any personal information, such as your name or address, with the vendors. If you have any questions, the study team can help you.*

*Meta (creater of Meta Quest Pro VR headset and Meta Horizon Worlds):*

[*https://www.facebook.com/terms.php*](https://www.facebook.com/terms.php)

[*https://www.facebook.com/privacy/policy*](https://www.facebook.com/privacy/policy)

*Roblox:*

[*https://en.help.roblox.com/hc/en-us/articles/115004647846-Roblox-Terms-of-Use*](https://en.help.roblox.com/hc/en-us/articles/115004647846-Roblox-Terms-of-Use)

[*https://en.help.roblox.com/hc/en-us/articles/115004630823-Roblox-Privacy-and-Cookie-Policy*](https://en.help.roblox.com/hc/en-us/articles/115004630823-Roblox-Privacy-and-Cookie-Policy)

1. Do you give permission to be contacted about future research?

Giving your permission for the Principal Investigator and sub-investigators on this study to contact you about future research studies is optional and voluntary. If you choose not to allow us to contact you, it will not affect your care at any of the NYU Langone Health facilities.

Please understand your permission is only for the purpose of helping us identify research participants who may qualify for one of our future research studies. It does not mean that you must join any study.

If you agree, then someone from Dr. Cassidy’s research staff might contact you in the future and tell you about an IRB-approved research study. At that time, you can decide whether or not you are interested in learning more about that particular study. You will then have the opportunity to contact the researcher to schedule an appointment to be fully informed about that particular research study.

____ I **agree** to be contacted by the Principal Investigator or sub-investigators regarding future IRB-approved research studies.

____ I **do not want** to be contacted by the Principal Investigator or sub-investigators regarding future IRB-approved research studies.

| **When you sign this form**, you are agreeing to take part in this research study as described to you. This means that you have read the consent form, your questions have been answered, and you have decided to volunteer. |
| --- |

|  |  |  |  |  |
| --- | --- | --- | --- | --- |
| Name of Participant (Print) |  | Signature of Participant |  | Date |
|  |  |  |  |  |
| Name of Person Obtaining Consent (Print) |  | Signature of Person Obtaining Consent |  | Date |

The study team has shared the true purpose of this research study with me during a debriefing interview and has provided a handout with all relevant details.

____ I **re-confirm my consent** to be part of this research study and to allow the Principal Investigator and study team to use my data as part of this research study.

____ I **no longer want** to be part of this research study and no longer consent to allow the Principal Investigator and study team to use my data as part of this research study.

Participant Signature: ________________________________

Date: __________


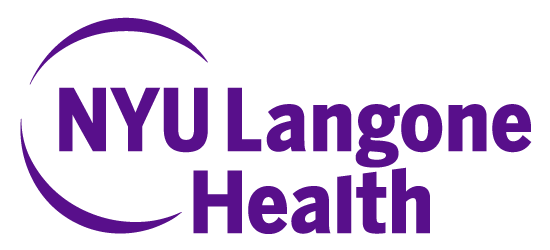
**Research Participant**

**Key Study Information Form**

| **Title of Study:** | YAES VR Studyi24-00910 |
| --- | --- |
| **Principal Investigator:** | Omni Cassidy, PhDDepartment of Population Health646-501-3546 |

You are being invited to take part in a research study being conducted by Dr. Omni Cassidy, Department of Population Health, at NYU Langone Health (NYULH). Your participation is voluntary which means you can choose whether or not you want to take part in this study.

**Purpose of the Research Study**

The purpose of this study is to understand more about young adults’ experiences and use of virtual reality (VR). In particular, we are interested in young adults’ feedback about new VR applications (“apps”). We are asking you to take part in this research study because you have said you are interested in participating in a study to share your thoughts about VR apps. You are eligible to participate because you are between 18-24 years old; self-identify as Black or white; self-report normal or corrected-to-normal vision from glasses or contacts; self-report comfort and ability to walk around within a VR environment; are able to read, write, understand, and respond to all study materials in English; reside in the NYC metropolitan area; are healthy; and have the capacity and willingness to provide consent.

**Other Key Information**

Participation in this study will involve one in-person study visit at 180 Madison Avenue, New York, NY (midtown Manhattan, NY) that will last about two hours. While in this study, you will be asked to:

- play one of two VR game apps while wearing VR headset that will go over your eyes
- give us saliva samples from your mouth
- place a sensor on your hand that will give us information about how you may be feeling in the moment
- complete questionnaires that include questions about yourself including, but not limited to, your level of education, race/ethnicity, age, and others
- have your body weight measured

**Foreseeable Risk and Benefits**

A comprehensive list of all possible risks and discomforts related to this research is included in the full consent. The most common risk experienced include experiencing motion sickness while wearing the VR headset, which is referred to as cybersickness.

You will not benefit personally from being in this study. However, we hope that, in the future, other people might benefit from this study because it will help us have a deeper understanding of the experiences young adults have with VR.

**Alternatives to Participation**

Should you choose not to participate, there are no alternatives besides not participating.

For in-depth details regarding this study, please refer to the full informed consent document attached.

For questions and concerns regarding any of this information, contact Omni Cassidy, PhD, Principal Investigator, at omni.cassidy@nyulangone.org.
