# Supplementary material for: Examining the Effect of Virtual Reality–Based Fast-Food Marketing on Eating-Related Outcomes in Young Adults: Protocol for a Randomized Controlled Trial
Source: JMIR Res Protoc. 2025 Sep 22;14:e69096. doi: 10.2196/69096 (PMC12501532; doi:10.2196/69096)
Supplement: Multimedia Appendix 4 [file resprot_v14i1e69096_app4.pdf]

SUMMARY STATEMENT
(Privileged Communication)

PROGRAM CONTACT:
CARLOS Garrido
(301) 402-1366
carlos.garrido@nih.gov

Release Date: **07/24/2024**
Revised Date:

Application Number: **1K01MD019320-01A1**
Formerly: **1K01MD019320-01**

Principal Investigator

CASSIDY, OMNI

Applicant Organization: NEW YORK UNIVERSITY SCHOOL OF MEDICINE

Review Group:	**ZMD1 DRI (O1)**<br>**National Institute on Minority Health and Health Disparities Special Emphasis Panel**<br>**NIMHD Mentored Career and Research Development Awards (Ks)**
Meeting Date:	**06/12/2024**
Council:	**OCT 2024**
Requested Start:	**09/01/2024**
Opportunity Number:	**PA-20-176**
PCC:	**IBB02CG**

Project Title:	**The effect of virtual reality-based food marketing on biological hunger signals and purchase intention in young adults**
SRG Action:	**Impact Score:30**
Next Steps:	**Visit https://grants.nih.gov/grants/next_steps.htm**
**Human Subjects:**	**30-Human subjects involved - Certified, no SRG concerns**
**Animal Subjects:**	**10-No live vertebrate animals involved for competing appl.**
**Gender:**	**1A-Both genders, scientifically acceptable**
**Minority:**	**1A-Minorities and non-minorities, scientifically acceptable**
**Age:**	**7A-Only Adults, scientifically acceptable**

Project Year	[redacted]	[redacted]
1	[redacted]	[redacted]
[redacted]	[redacted]	[redacted]

ADMINISTRATIVE BUDGET NOTE: The budget shown is the requested budget and has not been adjusted to reflect any recommendations made by reviewers. If an award is planned, the costs will be calculated by Institute grants management staff based on the recommendations outlined below in the COMMITTEE BUDGET RECOMMENDATIONS section.

1K01MD019320-01A1 Cassidy, Omni

BIOHAZARD COMMENT

RESUME AND SUMMARY OF DISCUSSION: This K01 application is re-submitted by New York University School of Medicine in response to PA-20-176, Mentored Research Scientist Development Award (Parent K01 - Independent Clinical Trial Required), on behalf of candidate Omni Cassidy, PhD. The resubmitted application has multiple strengths, addresses an important public health issue, and is considered very responsive to prior critiques. This outstanding candidate is a population health researcher and tenure-track Assistant Professor in the Section on Health Choice, Policy, and Evaluation in the Department of Population Health, with prior relevant training and experience (e.g., F31, T32) and evidence of productivity (honors, presentations, publications). The area of independent research addresses the use of advanced digital technologies (e.g., virtual reality, artificial intelligence, machine learning) to influence consumer behavior, with a specific interest in the impact of immersive food and beverage marketing on Black communities and further plans for monitoring health impacts and leveraging technology to inform policy and support nutritional equity. The career development plan describes a strong training component, that includes virtual reality (VR) training at Aspen Institute, integrating biological measurement with a VR paradigm, advanced statistical analyses, and strategies for informing/influencing food policy. The research plan's focus on racialized marketing as a public health issue is considered innovative and timely. Other strengths include the use of a use of multi-method approach and community advisory board (CAB). The mentorship team is exceptional with the inclusion of leading experts in relevant fields. The research environment is exceptional as well with resources for specialization in VR technology. There is strong evidence of institutional commitment to and support for the candidate, including 75 percent protected time. Some weaknesses are noted. Training goals are somewhat generic and unfocused, which seems a missed opportunity for learning from experts (e.g., interface of technology impacts on behavior, biological responses to food, identification of relevant measurements/metrics for use with the multi-level model). The biological training proposed seems mostly anatomy based with minimal training regarding the impact of food security/insecurity. While a conceptual model has been added, concerns remain given that mediation in the model is unclear. Additional minor concerns regarding the research plan include the need for more clarification regarding planned recruitment; the role of food insecurity is unaddressed; no clear plan for handling/storing biosamples; and lack of detail about CAB involvement. Finally, data collection continues through Year 5, but preparation of the R01 begins in Year 3. Overall, this is an excellent application with some minor weaknesses and the potential for high impact.

DESCRIPTION (provided by applicant): This Mentored Research Scientist Development (K01) Award will support my long-term goal of becoming an independent investigator who specializes in examining the techniques food companies use to market unhealthy products to Black communities, with a specific focus on advanced digital technologies, such as virtual reality (VR). Food marketing has been unequivocally linked to poor diet and risk of excessive weight gain in young people. Food companies disproportionately target Black consumers with more advertisements ("ads") and the least healthy ads, contributing to nutritional disparities and nutrition-related diseases. To expand their reach, food companies have developed VR marketing experiences that are engaging, highly sophisticated, and may more powerfully elicit biological signals precipitating a motivation to consume foods. Although most food marketing studies focus on children and adolescents due to their inability to recognize persuasive intent, Black young adults may be especially vulnerable to the effects of food marketing exposure due to being highly targeted, shifts during this developmental period, and food/nutritional insecurity. No study has examined the effect of VR food marketing exposure on biological signal activation and purchase intention among Black young adults, or the interactions with food/nutritional insecurity. To address these gaps, I have developed the following specific aims/hypotheses. Aim 1 will be to determine the extent to which VR food marketing influences purchase intention and whether effects are more pronounced in Black young adults. I hypothesize that exposure to VR food marketing will

influence purchase intention versus the VR non-food control, and Black (versus white young adults) exposed to VR food marketing will demonstrate higher purchase intention. Aim 2 will be to understand the influence of biological signal activation on the relationship between VR food marketing and purchase intention. I hypothesize that larger electrodermal amplitude and higher salivary reactivity will lead to stronger purchase intention in those randomized to VR food marketing versus the control and that the effects will be more pronounced among those with higher weight. Aim 3 will be to explore the interactions among race/ethnicity, food/nutritional insecurity, biological signal activation, and purchase intention among Black young adults exposed to VR food marketing. I hypothesize that those reporting low/very low food security will demonstrate stronger electrodermal amplitude and higher salivary reactivity when exposed to the VR food marketing condition versus all others. I will develop a community-based young adult advisory board to assist with interpretation of the results. It will be necessary to obtain mentorship and training in VR, integrating biological measurements into an experimental paradigm, and applied statistical analyses for biological outcomes to complete this project and develop the critical research competencies to elucidate how advanced digital food marketing strategies influence nutritional disparities and nutrition-related disease risk among Black young adults. Findings can inform novel behavioral interventions and regulatory efforts, such as the Federal Trade Commission's initiative to reduce stealth digital food marketing targeting vulnerable groups.

PUBLIC HEALTH RELEVANCE: The goal of this proposal is to elucidate the effect of unhealthy food and beverage marketing as a key environmental determinant that places Black young adults at increased risk for nutritional disparities and nutrition-related chronic diseases, including type 2 diabetes, hypertension, and some cancers. The proposed research will evaluate the extent to which exposure to virtual reality (VR)-based food marketing influences biological signals (i.e., electrodermal activity and salivation) and purchase intention following a lab-based randomized experiment in a sample of Black and white young adults. Findings can inform novel behavioral interventions and current regulatory efforts, such as the Federal Trade Commission's initiative to reduce stealth digital food marketing targeting vulnerable groups.

CRITIQUE 1

Candidate: 2
Career Development Plan/Career Goals /Plan to Provide Mentoring: 3
Research Plan: 2
Mentor(s), Co-Mentor(s), Consultant(s), Collaborator(s): 1
Environment Commitment to the Candidate: 1

OVERALL IMPACT: This resubmitted K01 application from an outstanding candidate proposes a research project and training to support the development of an innovative research program addressing immersive food marketing targeting Black communities. Dr. Cassidy has a solid foundation for conducting the proposed work, including experience in community-based participatory research (CPBR), food marketing in Black communities, and virtual reality (VR) technologies, and has a history of funding for her research. Her detailed training plan will build on her prior training and expand her skill set to include biological measurements relevant to food marketing research and accompanying advanced statistical techniques, with strong support from an exceptional team of mentors. New York University Grossman School of Medicine is an ideal environment for the proposed research and training. The project addresses a critical public health issue in a vulnerable population, with a timely and innovative approach, soliciting input from the target population through engagement of a community advisory board. Minor weakness in Dr. Cassidy's career development plan, such as the absence of clinical trials specific training and limited hands-on training in biological responses to food

and their measurement, and some lack of clarity in the study design do not substantially dampen enthusiasm for this application, which has high potential impact.

1. CANDIDATE

Strengths

- Dr. Cassidy has been the recipient of an F31, a grant from Duke University for a visiting scholar position, and an NSF summer fellowship.
- Her training and research experiences, including conducting an experiment on racially-targeted food marketing for her F31, coordinating a P20 project involving cultural adaptation of an RCT through community-based participatory research (CPBR), and learning content analysis as part of her postdoctoral fellowship, provide an ideal foundation for the proposed project and career development activities.
- Dr. Cassidy's letters of support are exceptionally strong.

Weaknesses

- Minor – Although she has published extensively in this area, only six of her publications are as first author.

2. CAREER DEVELOPMENT PLAN/CAREER GOALS AND OBJECTIVES

Strengths

- The training plan builds on her prior experience, providing clearly articulated training needs for her long-term career objective of conducting policy-informing research on immersive food marketing targeting Black communities.
- Her plan is detailed and involves an appropriate frequency of contact with members of her mentoring team.
- The proposed training in particular in VR is comprehensive.
- The Aspen Institute Food Leaders Fellowship is also an excellent training activity for advancing her knowledge of food systems and policy.

Weaknesses

- Dr. Cassidy has relatively little experience with clinical trials and no formal training is planned.
- Her planned activities for her training in biological responses to food and their measurement are largely didactic and lab attendance, with minimal hands-on experiences.

3. RESEARCH PLAN

Strengths

- The project tackles the important public health issue of racially targeted food marketing, which negatively impacts health in Black communities, using innovative technology.
- The focus on VR is timely and well suited to young adults.
- Young adulthood can be a particularly vulnerable period with respect to food choices, given the transition to independent living/meal preparation and limited disposable income.
- The integration of biological measures and consideration of food insecurity provide a complex characterization of VR marketing effects.

- A community advisory board will provide input and feedback on the study.
- A power analysis supporting the statistical plan is provided.

Weaknesses

- Representation of individuals across weight status (e.g., underweight, obesity) is not discussed.
- The debriefs/semi-structured interviews will be coded, but it is unclear how these data will be used.
- There is no mention of how prior experience with Wendyverse or Nikeland might impact eligibility or be considered in analyses.

4. MENTOR(S), CO-MENTOR(S), CONSULTANT(S), COLLABORATOR(S)

Strengths

- Dr. Cassidy has assembled a stellar mentoring team of experts in their respective areas of research and strong funding records, and most have extensive mentoring experience.
- The team collectively possesses expertise in all relevant training areas: food marketing and purchasing behaviors, VR, biological measures in food marketing research, statistical approaches with biological outcomes, and community engagement, including community advisory board formation.
- The mentoring team members express strong support for Dr. Cassidy's project and career development.

Weaknesses

- None noted.

5. ENVIRONMENT AND INSTITUTIONAL COMMITMENT TO THE CANDIDATE

Strengths

- New York University Grossman School of Medicine is an exceptional environment for conducting the proposed research and training activities. Supporting resources include The Institute for Innovations in Medical Education (VR and other advanced technologies), the health equity-centered Department of Population Health's partnerships with communities, and the Community Engagement & Population Health Research Program.
- The letter from the Department Chair expresses extremely strong institutional support.

Weaknesses

- None noted.

Study Timeline

Strengths

- The timeline is detailed and feasible.

Weaknesses

- None noted.

Protections for Human Subjects

Acceptable Risks and Adequate Protections

- Appropriate measures are in place to store data securely. Minimal potential risks associated with the VR protocol are addressed, including limiting VR paradigms to 15 minutes and excluding individuals with a high propensity for motion sickness.

Data and Safety Monitoring Plan (Applicable for Clinical Trials Only):

Acceptable

- The PI, mentors, and study staff will review adverse events and monitor protocol adherence.

Inclusion Plans

- Sex/Gender: Distribution justified scientifically
- Race/Ethnicity: Distribution justified scientifically
- For NIH-Defined Phase III trials, Plans for valid design and analysis: Not applicable
- Inclusion/Exclusion Based on Age: Distribution justified scientifically
  - The proposed age range and racial/ethnic composition of the study sample are appropriate. A diverse sample (with respect to gender, ethnicity, and other social identities) representative of the target population (i.e., Black and White young adults) will be recruited.

Vertebrate Animals

Not Applicable (No Vertebrate Animals)

Biohazards

Not Applicable (No Biohazards)

Resubmission

- Dr. Cassidy has been very responsive to the prior critiques. She has made substantial changes and adequately addressed the critiques.

Training in the Responsible Conduct of Research

Acceptable

Comments on Format (Required):

- Training will consist of coursework, workshops, and meetings with mentors.

Comments on Subject Matter (Required):

- Trainings will cover relevant content, including privacy and information security, compliance. No training specific to clinical trials is described.

Comments on Faculty Participation (Required; not applicable for mid- and senior-career awards):

- Dr. Cassidy will meet one-on-one with mentors.

Comments on Duration (Required):

- Training will start in Year 1 and continue throughout period of award.

Comments on Frequency (Required):

- Meetings with mentors will occur as frequently as bi-weekly. Formal coursework and workshops will vary in frequency.

Select Agents

Not Applicable (No Select Agents)

Resource Sharing Plans

Not Applicable (No Relevant Resources)

Authentication of Key Biological and/or Chemical Resources

Not Applicable (No Relevant Resources)

Budget and Period of Support

Recommend as Requested

CRITIQUE 2

Candidate: 2
Career Development Plan/Career Goals /Plan to Provide Mentoring: 5
Research Plan: 4
Mentor(s), Co-Mentor(s), Consultant(s), Collaborator(s): 1
Environment Commitment to the Candidate: 1

OVERALL IMPACT: The application strives to support the candidate's career goals of studying food and beverage marketing that uses advanced digital technologies. A mix of didactive and mentor-driven development activities are proposed, along with research aims focused on understanding the relationships between virtual-reality food marking exposure, biological responses, behavioral responses, and race. Key strengths include a candidate with relevant training and experiences, exceptional mentors and environment, and an innovative focus on potentially emergent topic as a public health concern. Key weaknesses include several underdeveloped career development goals; confused relationships within the conceptual model, hypotheses, and analysis plan; and resulting analytic approaches that appear to be inappropriate for the knowledge gain sought. On balance, this yields an assessment that the proposed career development and research plan will have a medium likelihood of enhancing the candidate's potential for a productive, independent health disparities research career.

1. CANDIDATE

Strengths

- The candidate has experience with food-ad-related lab experimental design and execution.
- The candidate has received grant funding through smaller mechanisms (F31, Duke University, CEHRT pilot).
- The candidate has supervisions experience and has established an early commitment to mentorship.

- The candidate has translated lived experience in a community negatively affected by the studied health disparities into a commitment to producing policy-relevant research.
- The candidate has experience conducting CBPR.

Weaknesses

- While the candidate has 5 first-authored papers conducting primary data collection, only two are within the last 6 years.

2. CAREER DEVELOPMENT PLAN/CAREER GOALS AND OBJECTIVES

Strengths

- The training plan includes a mix of coursework, mentor meetings, and workshop/conference settings.
- The webinar listed under training goal 3 is specific, supports an identified gap, and will aid the candidate in completing proposed research activities.
- The Aspen Institute Food Leaders Fellowship is highly relevant for training goal 4 and connects to the candidate's broader goals to formulate policy-relevant research.

Weaknesses

- For training goal 1, it is unclear what the specific gaps related to VR need to be addressed. Relatedly, the training activities focus on general topics related to various digital technologies (each of which are independently complex) rather than specifically targeted subject matter or methodological content that will clearly support the candidate's goals. A Co-Mentor, Dr. Persky, appears to be a very strong mentor in this area, and the candidate and this mentor will have regular meetings in Years 1, 4, and 5 (unclear why not in Years 2 and 3, or why in Years 4/5 vs. 2/3). However, there appears to be missed opportunities for not better leveraging this mentor and the Immersive Simulate Program the mentor leads for more focused training activities in this domain.
- For training goal 2, it is unclear why the main coursework is dedicated to theory vs. measurement, given that the candidate possesses a background on biological systems but has identified a gap in measurement. The virtual attendance at research lab meetings is a strength that reduces the weight of this weakness, however.
- Given Aim 3 and the goal of preparing an R01 submission, some training specific to food insecurity is needed and perhaps could be added to training goal 4.

3. RESEARCH PLAN

Strengths

- Nutrition quality disparities are an important public health topic.
- Disparities in food advertisement volume and type are an important exposure for study as a potential point of intervention.
- Studying VR food marketing as an emerging and potential public health exposure risk is innovative.
- Alternative scenarios are presented to address situations in which the stated plan runs into obstacles.

Weaknesses

- The main concern is that the relationships between the hypotheses, conceptual model, and methods are unclear, as highlighted in the points below. This lack of clarity makes the overall direction of the research questions unclear, lessening the likelihood that the research activities will support the candidate's proposed career goals.
- It appears that Aim 2 is structured as a moderation analysis when it should be structured as a mediation analysis. Under the proposed conceptual model, food cues in marketing should activate biological responses, ultimately leading to behavioral responses; this describes a mediation model in which biological responses mediate the relationship between ad exposure and food-related decisions. However, the analyses and hypotheses (#'s 3 and 4) are structured to examine moderation as the mechanism. Moderation would imply that the relationship between food cues and behavior differ among those with different levels of biological reactivity. This significance section motivates the case that Black young adults may have greater reactivity to food marketing, and perhaps this led to the moderation analysis. However, with that motivation, the natural analysis would still be the mediation approach, with race acting as a moderator heightening the strength of the associations between ad exposure and biological reactions (given previous exposures to structural racism).
- Relatedly, it is unclear why the analytic approach for Aim 3 does not include interactions with the VR exposure variable. As in the point above, if food insecurity (FI) exposure leads to heightened sensitivity to ads via biological responsiveness, then again it is a mediation model that is of interest—only this time the moderation variable would be food insecurity (or, presumably in a more exploratory analysis given power constraints: FI, race, and their interaction would all be moderators, which would be modeled as a triple interaction between FI, race, and ad exposure).
- It is unclear how the power analysis for Aim 3 accounts for the hypothesized interaction.
- No background information related to food insecurity and advertisement exposure/sensitivity is provided.

4. MENTOR(S), CO-MENTOR(S), CONSULTANT(S), COLLABORATOR(S)

Strengths

- The candidate has an ongoing mentoring relationship with the primary mentor, and they have collaborated on publications and grant submissions.
- Co-mentor Boyland provides relevant expertise related to the targeted biology-related career development goals.
- Co-mentor Persky provides relevant expertise related to VR experimental lab research.
- Co-mentors Troxel and Islam also will provide relevant biostatistical and CBPR-related support.
- Mentors all have relevant mentorship experience.

Weaknesses

- None noted

5. ENVIRONMENT AND INSTITUTIONAL COMMITMENT TO THE CANDIDATE

Strengths

- The candidate has access to adequate laboratory space and wealth of resources through the Section of the primary mentor and the overall academic medical center.

- Institutional commitment protecting 75 percent FTE is provided; ongoing mentorship and startup funds also indicate strong support.

Weaknesses

- None noted.

Study Timeline

Strengths

- Candidate has experience recruiting participants for lab-based ad-exposure experiments, increasing the likelihood of being able to successfully adhere to the proposed timeline.

Weaknesses

- There is some tension between developing an R01 in Year 3 while still collecting the data that would presumably be needed as preliminary data for the R01 through Year 5, although it is likely that some data will be available earlier.

Protections for Human Subjects

Acceptable Risks and Adequate Protections

- No concerns

Data and Safety Monitoring Plan (Applicable for Clinical Trials Only):

Acceptable

- o No concerns

Inclusion Plans

- Sex/Gender: Distribution justified scientifically
- Race/Ethnicity: Distribution justified scientifically
- For NIH-Defined Phase III trials, Plans for valid design and analysis: Not applicable
- Inclusion/Exclusion Based on Age: Distribution justified scientifically
  - o No concerns

Vertebrate Animals

Not Applicable (No Vertebrate Animals)

Biohazards

Not Applicable (No Biohazards)

Resubmission

- The candidate has added a conceptual model, which has some strengths to the significance section. However, it is still fairly rudimentary, and references that it is guided by other frameworks, such as the socio-ecological model, while not actually describing what this guidance wis or how it shapes the conceptual model presented.

- The candidate does not appear to have addressed the comment about inadequate detail regarding the incorporation of the CAB.
- The aims have been restructured considerably in light of the comments in the summary statement; details about policy relevance have been provided.

Training in the Responsible Conduct of Research

Acceptable

Comments on Format (Required):

- No concerns.

Comments on Subject Matter (Required):

- No concerns.

Comments on Faculty Participation (Required; not applicable for mid- and senior-career awards):

- No concerns.

Comments on Duration (Required):

- No concerns.

Comments on Frequency (Required):

- No concerns.

Select Agents

Not Applicable (No Select Agents)

Resource Sharing Plans

Not Applicable (No Relevant Resources)

Authentication of Key Biological and/or Chemical Resources

Not Applicable (No Relevant Resources)

Budget and Period of Support

Recommend as Requested

- No concerns.

CRITIQUE 3

Candidate: 2
Career Development Plan/Career Goals /Plan to Provide Mentoring: 2
Research Plan: 3
Mentor(s), Co-Mentor(s), Consultant(s), Collaborator(s): 2
Environment Commitment to the Candidate: 1

OVERALL IMPACT: The training plan aims to further training of a promising early career researcher interested in research geared focused on reducing disparities unhealthy eating. The candidate’s

experience thus far is a good indicator of success of the proposed training plan and research project given her prior experiences (including publications and other research experiences). The plan is consistent with the candidate's stated career and training goals and she has assembled a team of experts to assist in that training. The proposed research plan is innovative and timely and based on a review of rigorous research. The mentoring team brings complementary expertise to aid in the training plans success. Most weaknesses are minor, but given the nature of them (e.g., monitoring of recruitment of participants based on race and/or sex, handling of biological samples) are important to address prior to award period.

1. CANDIDATE

Strengths

- Candidate appears to have the experience necessary to complete the proposed project, including a record of publications in this area.
- Candidate has published 26 papers, 5 of which are as first author, and has experience as PI on funded projects.

Weaknesses

- None noted.

2. CAREER DEVELOPMENT PLAN/CAREER GOALS AND OBJECTIVES

Strengths

- Candidate's long-term career goal is to build an independent research program within my newly developed Food, Culture, & Tech Lab to monitor the use and effects of unhealthy food and beverage marketing that utilizes advanced digital technologies, particularly virtual reality (VR). She has an explicit focus on promoting health equity.
- Training appears to meet candidate's training and career goals.
- Training goals include training in VR and health, biology of food and eating, and food systems and eating.
- Training will be a mix of coursework, mentor meetings that include directed readings, conference attendance, and mentored experiential training.

Weaknesses

- None noted.

3. RESEARCH PLAN

Strengths

- Multi-method approach is a strength.
- Aims appear informed by prior rigorous research.
- The use of VR is innovative.
- Given increasing rates of obesity and other unhealth eating, especially in the Black community, the study is timely with important health-related implications.
- Conceptual framework includes individual, biological, social, environmental, and behavioral elements.
- Involvement of community advisory board another strength.

Weaknesses

- Application lacks plan to monitor recruitment and other strategies to ensure near-equal numbers of Black and White participants.

4. MENTOR(S), CO-MENTOR(S), CONSULTANT(S), COLLABORATOR(S)

Strengths

- Dr. Elbel will provide mentorship on food related research. He has experience mentoring early career researchers.
- Dr. Boyland will provide mentorship on biological mechanism measurement. She also has experience mentoring early career researchers.
- Dr. Persky will mentor the VR component.
- Dr. Triola will also provide mentorship in VR.
- Dr. Troxel will provide mentorship in clinical trial design. She also has experience mentoring early career researchers.

Weaknesses

- The application lacks details regarding the unique contributions of Drs. Persky and Triola.

5. ENVIRONMENT AND INSTITUTIONAL COMMITMENT TO THE CANDIDATE

Strengths

- The NYU Grossman School of Medicine seems to have the resources necessary to support this project.

Weaknesses

- None noted.

Study Timeline

Strengths

- Seems appropriate.

Weaknesses

- None.

Protections for Human Subjects

Acceptable Risks and Adequate Protections

- Although all sources of data will be labeled with ID to protect confidentiality, the application lacks a discussion regarding how salivary samples will be handled.

Data and Safety Monitoring Plan (Applicable for Clinical Trials Only):

Acceptable

- o PI will monitor and report any adverse effects.

Inclusion Plans

- Sex/Gender: Distribution not justified scientifically
- Race/Ethnicity: Distribution justified scientifically
- For NIH-Defined Phase III trials, Plans for valid design and analysis: Not applicable
- Inclusion/Exclusion Based on Age: Distribution justified scientifically
    - Age range from 18- to 25-years-old is justified.
    - Selection of both Black and White sample is justified.
    - Although women will be included, the application lacks discussion about how many; and provides no apparent rationale for the proposed sampling frame concerning women and strategies for ensure representation of more than one sex.

Vertebrate Animals

Not Applicable (No Vertebrate Animals)

Biohazards

Unacceptable

- The application lacks any discussion of the safe handling/storage of salivary samples.

Resubmission

- The application is responsive to initial reviews.

Training in the Responsible Conduct of Research

Unacceptable

Comments on Format (Required):

- CITI renewals, mentorship meetings, courses, seminars, workshops.

Comments on Subject Matter (Required):

- New Submissions; Reportable New Information; Continuations, Modifications, and Closures; How to Write a Protocol; Writing an Informed Consent that Informs; Informed Consent Process; and How to Keep a Regulatory Binder.
- The application lacks plans for training in research with underserved groups, biological specimen, or VR.

Comments on Faculty Participation (Required; not applicable for mid- and senior-career awards):

- The candidate will teach a course.
- The involvement of faculty mentors in these training is not stated explicitly.

Comments on Duration (Required):

- 8 contact hours.

Comments on Frequency (Required):

- First, second, and third Mondays of every month.

Select Agents

Not Applicable (No Select Agents)

Resource Sharing Plans

Not Applicable (No Relevant Resources)

Authentication of Key Biological and/or Chemical Resources

Not Applicable (No Relevant Resources)

Budget and Period of Support

Recommend as Requested

THE FOLLOWING SECTIONS WERE PREPARED BY THE SCIENTIFIC REVIEW OFFICER TO SUMMARIZE THE OUTCOME OF DISCUSSIONS OF THE REVIEW COMMITTEE, OR REVIEWERS' WRITTEN CRITIQUES, ON THE FOLLOWING ISSUES:

PROTECTION OF HUMAN SUBJECTS: ACCEPTABLE

INCLUSION OF WOMEN PLAN: ACCEPTABLE

INCLUSION OF MINORITIES PLAN: ACCEPTABLE

INCLUSION ACROSS THE LIFESPAN PLAN: ACCEPTABLE

BIOHAZARD COMMENT:
The application lacks any discussion of the safe handling/storage of salivary samples.

COMMITTEE BUDGET RECOMMENDATIONS: The budget was recommended as requested.
NOTE: The budget does not appear to include collection or storage costs related to biospecimens.

RESPONSIBLE CONDUCT OF RESEARCH:
The application lacks plans for training in research with underserved groups, biological specimen, or virtual reality.

Footnotes for 1K01MD019320-01A1; PI Name: Cassidy, Omni

NIH has modified its policy regarding the receipt of resubmissions (amended applications).See Guide Notice NOT-OD-18-197 at https://grants.nih.gov/grants/guide/notice-files/NOT-OD-18-197.html. The impact/priority score is calculated after discussion of an application by averaging the overall scores (1-9) given by all voting reviewers on the committee and multiplying by 10. The criterion scores are submitted prior to the meeting by the individual reviewers assigned to an application, and are not discussed specifically at the review meeting or calculated into the overall impact score. Some applications also receive a percentile ranking. For details on the review process, see http://grants.nih.gov/grants/peer_review_process.htm#scoring.

MEETING ROSTER

The roster for this review meeting is displayed as an aggregated roster that includes reviewers from multiple MD Special Emphasis Panels Meetings
for the 2024/10 council round.
This roster for MD is available here.
